# The Structural and Functional Basis of Catalysis Mediated by NAD(P)H:acceptor Oxidoreductase (FerB) of *Paracoccus denitrificans*

**DOI:** 10.1371/journal.pone.0096262

**Published:** 2014-05-09

**Authors:** Vojtěch Sedláček, Tomáš Klumpler, Jaromír Marek, Igor Kučera

**Affiliations:** 1 Department of Biochemistry, Faculty of Science, Masaryk University, Brno, Czech Republic; 2 Central European Institute of Technology, Masaryk University, Brno, Czech Republic; Oak Ridge National Laboratory, United States of America

## Abstract

FerB from *Paracoccus denitrificans* is a soluble cytoplasmic flavoprotein that accepts redox equivalents from NADH or NADPH and transfers them to various acceptors such as quinones, ferric complexes and chromate. The crystal structure and small-angle X-ray scattering measurements in solution reported here reveal a head-to-tail dimer with two flavin mononucleotide groups bound at the opposite sides of the subunit interface. The dimers tend to self-associate to a tetrameric form at higher protein concentrations. Amino acid residues important for the binding of FMN and NADH and for the catalytic activity are identified and verified by site-directed mutagenesis. In particular, we show that Glu77 anchors a conserved water molecule in close proximity to the O2 of FMN, with the probable role of facilitating flavin reduction. Hydride transfer is shown to occur from the 4-*pro*-*S* position of NADH to the solvent-accessible *si* side of the flavin ring. When using deuterated NADH, this process exhibits a kinetic isotope effect of about 6 just as does the NADH-dependent quinone reductase activity of FerB; the first, reductive half-reaction of flavin cofactor is thus rate-limiting. Replacing the bulky Arg95 in the vicinity of the active site with alanine substantially enhances the activity towards external flavins that obeys the standard bi-bi ping-pong reaction mechanism. The new evidence for a cryptic flavin reductase activity of FerB justifies the previous inclusion of this enzyme in the protein family of NADPH-dependent FMN reductases.

## Introduction

There is an expanding list of bacterial flavoenzymes that display redox activities against diverse xenobiotics, such as heavy metal ions like chromate [1], nitro compounds [2], azo dyes [3] and carbonyl compounds including quinones [4]. These enzymes occur as homodimers or homotetramers with a non-covalently bound flavin mononucleotide (FMN) cofactor and display a bi-bi ping-pong mechanism with NAD(P)H as the presumed physiological reductant. Despite a progress in elucidating high-resolution structures of some representatives and understanding the underlying flavin redox chemistry, key questions remain still open, in particular as regards the identity of natural substrates and the true significance in bacterial metabolism.

The flavoprotein FerB (ferric reductase B, a product of gene 4071) was originally isolated from the cytoplasm of the soil bacterium *Paracoccus denitrificans* as one of two major enzymes able to reduce Fe(III)-ligand complexes when NADH was the electron donor [5]. The protein is also active as a chromate reductase [5], [6] and, to a substantially greater extent, as a quinone reductase [5], [7]. Among the soluble quinone substrates tested, 2,3-dimethoxy-5-methyl-1,4-bezoquinone (ubiquinone-0, UQ-0) yielded the highest *k*_cat_/*K_m_* specificity ratio, suggesting that FerB may, in vivo, interact with benzoquinone ring of UQ-10, a lipophilic electron carrier abundantly present in the cytoplasmic membrane of the bacterium.

An initial Pfam domain analysis [7] placed FerB within the protein family of NADPH-dependent FMN reductases (Pfam database accession number PF03358). Some members of this family contain by themselves a non-covalently bound flavin cofactor (as does FerB) and are in fact quinone reductases, catalyzing two-electron reduction of quinones to respective hydroquinones [4]. Since modulation of the intracellular activity of quinone reductases in many instances affects cell responsiveness to redox stressors, these enzymes are believed to serve as protective devices for preventing semiquinone formation and subsequent free radical production or for quinone-mediated quenching of H_2_O_2_ [8]. However, additional roles beyond detoxification are not excluded as well. For example, there is evidence that eukaryotic quinone reductases associate with the 20S proteasome in redox state dependent manner and control in this way proteasomal degradation of some transcription factors [9]. From the practical point of view the substrate specificity towards metal ions makes these enzymes good candidates for biodegraders of highly toxic inorganic contaminants such as chromate or uranyl [10]. They may also find therapeutic application in prodrug processing by reductive enzymatic activation [11].

In this study, our main goal is to identify the amino acid residues of FerB responsible for flavin cofactor binding and/or catalysis of its redox conversions. For this, the crystal structure of holoenzyme has been solved and the effect of mutating the strategically selected residues has been analyzed by means of fluorimetric titration of free flavin by apoenzyme, enzyme kinetic assays and spectropotentiometry. Although there are no direct structural data on the enzyme-NADH complex, novel pieces of information about the interactions of FerB with NADH are provided by the observed stereochemistry of hydride abstraction from NADH and computer docking analyses. We also show that a simple steric modulation of the active site enhances its ability to reduce external flavin, which is otherwise negligible for the wild-type enzyme.

## Materials and Methods

### Gene Cloning, Protein Expression and Purification

The cloning, expression, and purification of the FerB protein and its selenomethionine derivative with a C-terminal six-histidine tag (FerBHis_6_) in *E. coli* strain BL21(DE3) pLysS cells (Invitrogen) were carried out as described in the paper [12].

### Site-directed Mutagenesis

Site-directed mutagenesis was carried out as specified in the QuikChange II Site-Directed Mutagenesis Kit (Stratagene). The pET21-ferB plasmid served as a PCR template; the primers are listed in Table S1. After the mutagenesis protocol, the sequences of the new *ferB* constructs were verified by sequence analysis. The mutated plasmids were purified using the Plasmid Mini DNA Purification Kit (Qiagen) and transformed into the host expression strain *E. coli* BL21(DE3) pLysS (Invitrogen). Expression of the recombinant protein mutants and the purification procedure by Ni-IDA (GE Healthcare) chromatography were performed as described for the wild-recombinant type enzyme [13]. Correct folding of all mutant enzymes was confirmed by circular dichroism spectroscopy.

### Crystallization, X-ray Data Collection and Structure Determination

Crystallization, X-ray diffraction data collection and reduction of the native FerBHis_6_ and its selenomethionine derivative are described in [12]. The crystal structure of FerBHis_6_ protein was determined by multiple wavelength anomalous dispersion using selenium anomalous signal. Excellent data quality and resolution allow us to try to solve phase problem of FerBHis_6_ using the advanced 3W-MAD protocol of Auto-Rickshaw: the EMBL-Hamburg automated crystal structure determination platform [14]. The Auto-Rickshaw structure determinative resulted in almost complete model of Se-Met FerBHis_6_ homodimer containing 349 residues (out of 380) divided into 9 chains with *R*/*R_free_*  =  0.2144/0.2682. Detailed description of the Auto-Rickshaw strategy in this particular case is described in [15]. Missing fragments were manually built using Coot [16] and this model of FerBHis_6_ was re-refined against 1.40 Å diffraction data and rebuild using PDB-REDO algorithm [17]. Final model of FerB (PDB ID: 3U7R) contains 364 residues with *R*/*R_free_*  =  0.16065/0.17386.

### Structure Alignments

A search for sequence and structural analogy was done using Basic Logical Alignment Search Tool (BLAST) and DALI server [18]. Multiple alignment analysis and phylogenetic tree construction was done using the PHYLIP program.

### SAXS (Small-angle X-ray scattering)

The SAXS data were collected on the BioSAXS-1000, Rigaku at CEITEC (Brno, Czech Republic). Data were collected at 290.15 K with X-ray beam wavelength λ = 1.54 Å. Sample to detector (PILATUS 100K, Dectris Ltd.) distance was 0.4 m covering a scattering vector range from 0.008 to 0.65 Å^−1^. For buffer and sample one two-dimensional image was collected with an exposure time of 30 min per image. The sample was measured at three concentrations: 5.0, 2.5 and 1.25 mg/mL. The buffer was identical to the buffer of the last step of the protein purification. Data reduction and the buffer substraction were performed using SAXSLab, Rigaku. Subtracted data were normalized to the protein concentration and truncated to 0.2 Å^−1^. Evaluation of the solution scattering of the FerBHis_6_ crystal structure and the fitting to experimental data was performed by CRYSOL [19]. The oligomeric state of the protein was evaluated using OLIGOMER [20] where the form-factors files were created by FFMAKER using dimeric and tetrameric assembly of the FerB crystal structure, both programs from ATSAS package [21] SAXS. Data collection and scattering-derived parameters are summarized in Table S2.

### Preparation of Apoflavoproteins

Approximately 2 mg of purified FerBHis_6_ or its mutants in the buffer containing 50 mM sodium phosphate, 300 mM NaCl and 10 mM imidazole, pH 8.0 were applied onto a 5 mL pre-equilibrated Ni Sepharose 6 Fast Flow column (GE Healthcare). After loading the protein, the column was washed 20 mL of starting buffer. Protein-bound flavins were then removed by washing the column with 100 mL of the same buffer additionally with 4 M KBr and 4 M urea (pH 8.0). The apo-form of the protein (apoFerBHis_6_) was eluted by the buffer containing 50 mM sodium phosphate, 300 mM NaCl and 500 mM imidazole, pH 8.0 (buffer C).

### Analytical Size Exclusion Chromatography

0.1 mL of sample (0.5 mg/mL of proteins) was loaded onto Superose 12 (GE Healthcare) analytical and preparative column (30 cm×1 cm) equilibrated by 25 mM Tris-HCl (pH 7.4). The flow rate after sample application was 0.5 mL/min. Marker proteins were albumin (67 kDa), ovalbumin (43 kDa), chymotrypsinogen A (25 kDa) and ribonuclease A (13.7 kDa).

### DSC (Differential scanning calorimetry)

Calorimetric measurements were performed on a VP-DSC MicroCalorimeter (MicroCal) with the cell volume 0.5 mL. Data were collected from 25 to 95 °C at a heating rate of 60 °C per hour. Protein concentrations were adjusted to 2.5 mg/mL for FerBHis_6_ and 4.3 mg/mL for apoFerBHis_6_ in buffer C. All solutions were degassed before measurements. Reference baseline was obtained by buffer vs. buffer scan and subtracted from the measured data. The obtained data were analyzed using the Origin software (Microcal Software).

### Dissociation Constants

Dissociation constants of apoenzyme-flavin complexes were determined from fluorescence titration with excitation at 450 nm by adding of constant amount of the wild-type and mutant variants of apoFerBHis_6_ into 25 mM Tris-HCl buffer (pH 7.4) with 100 nM FMN or riboflavin. Decreasing emission intensities at 519 nm were recorded on Luminescence Spectrotometer LS-50B (Perkin-Elmer). Series of different concentrations of FMN standard was used for setting of a calibration curve on fluorescence intensity. The dissociation constants *K_d_* were calculated according to [22] and converted to the binding Gibbs energy changes Δ*G_b_*  =  -RTln (1/*K_d_*) (where R, gas constant, T, absolute temperature), from which the variation of Gibbs energy due to mutation (ΔΔ*G_b_*) was computed as the difference in binding energy between mutant and wild-type enzyme (ΔΔ*G_b_*  =  Δ*G_b_*(mut) - Δ*G_b_*(wt)).

### Steady-State Kinetics

The initial velocity of NADH oxidation was measured spectrophotometrically at 340 nm (ε = 6.22 mM^−1^ cm^−1^) after the addition of variable concentrations of NADH to the solution containing the enzyme and a fixed concentration of electron acceptor (UQ-0 or flavin) in 25 mM Tris-HCl (pH 7.4). The kinetic parameters ± standard error were determined by fitting the data to the appropriate kinetic equation using nonlinear regression and the program Microcal Origin (version 5.0).

### Determination of the Standard Redox Potential

The standard redox potentials of FerBHis_6_ and its mutants were determined at 25 °C according to the method of Massey [23]. The reaction mixture contained in 1-mL cuvette the appropriate amount of the protein, 40 µM Indigo Carmine or 15 µM phenosafranin, 0.4 mM xanthine, 5 µM benzylviologen in 50 mM sodium phosphate buffer with 0.1 mM EDTA (pH 7.0). After flushing by argon (99.9999%, (v/v)), a catalytic amount of milk xanthine oxidase (0.16 µM) was added to the mixture and the reduction of FerBHis_6_-bound FMN and the dye was recorded by UV-VIS spectrophotometer (UltraSpec 2000, GE Healthcare). For each time, the concentrations of oxidized and reduced FMN and dyes at 450 nm and 521 nm (phenosafranin) or 610 nm (Indigo Carmine) corrected for the contribution of the appropriate dye and FMN, were quantified.

### Rapid Kinetics Measurements

Stopped-flow spectrophotometry was performed on a SFM-3000 (Bio-Logic) stopped-flow system equipped with a 0.8- × 0.8-mm cuvette (FC-08). The temperature was kept constant at 10 °C with a thermostating water bath. Six measurements were averaged for each sample. In a typical run 75 µL of a 30 µM protein solution was mixed with 75 µl of 10 mM NADH. A flow speed of 7 ml/s resulted in a dead time of 0.4 ms. Results from the stopped-flow experiments were analyzed in the Bio-Kine software (BioLogic).

### Stereospecifity Determination

[4A-^2^H]NADH was prepared by mixing of 5.6 mM NAD^+^, 48 mM deuterated ethanol-d_6_ (99,5% D, Sigma), 25 units of horse-liver alcohol dehydrogenase, 17 units of yeast aldehyde dehydrogenase in 10 mL of 6 mM Bis-Tris/Propane Buffer (pH 9.0) at 25 °C. The pH was maintained at constant level of the buffer with additions of KOH. The end of the reduction was reached during 1-2 hours and was confirmed at 340 nm. The enzymes were removed on ice by ultrafiltration through YM3 (cut-off limit 3kDa) filters (Millipore). The temperature of the filtrate was decreased to −20 °C and [4A-^2^H]NADH was lyophilized by LyoQuest (Telstar). [4B-^2^H]NADH was prepared by mixing of 4.5 mM NAD^+^, 5 mM ATP, 6 mM deuterated D-glucose-1-d (98% D, Aldrich), 10 mM MgSO_4_, 3.5 units of yeast hexokinase and 4 units of glucose-6-phosphate dehydrogenase in 10 mL of 6 mM Tris-HCl buffer (pH 8.0) at 25 °C. The removing of the enzymes and lyophilization were described above. Enzymes and nucleotides were purchased from Sigma. The speciphically deuterium-labelled reduced coenzymes were reoxidized by FerBHis_6_. The total reaction volume was 1 mL of 50 mM sodium phosphate buffer (pH 7.4) and contained approximately 5 mM [4A-^2^H]NADH or [4B-^2^H]NADH, 10 mM 1,4-BQ (1,4-benzoquinone) and 5 µM enzyme. When there was no further decrease at 340 nm, the mixture was ultrafiltrated and lyophilized as was described above. The lyophilized samples of NAD^+^ were dissolved in deuterium oxide (99.8%, Sigma) and all NMR spectra were measured at 25 °C on a Bruker Avance 500 MHz spectrometer (Bruker). The control spectra of 10 mM 1,4-BQ and 1,4-BQH_2_ were also recorded and no interference was observed in the area of the H-4C resonance signal. Chemical shifts are expressed as p.p.m. relative to trimethylsilan.

### In-silico Docking

*AutoDock Vina v1.1.2.* was used as a docking tool to generate putative NAD^+^/FerBHis_6_ complex. Input files were prepared using the *Autodock Tools* where Gasteiger charges were assigned to a ligand and receptor and subsequently non-polar hydrogens were merged. The FerBHis_6_ receptor was set as rigid during docking and the grid box with dimensions of 15 × 15 × 15 Å was placed into the NAD^+^ binding pocket. Exhaustiveness of the search was set to the value of 20 while all other *AutoDock Vina* parameters were kept as default. For FMN/FerBHis_6_ complex generation, the FMN coordinates were removed from the FerBHis_6_ receptor file. The grid box was with dimensions of 40 × 40 × 40 Å was placed into the FMN binding pocket, the receptor was set as rigid and all other *AutoDock Vina* parameters were kept as default.

## Results

### Crystal Structure of FerBHis_6_

**Figure 1:**
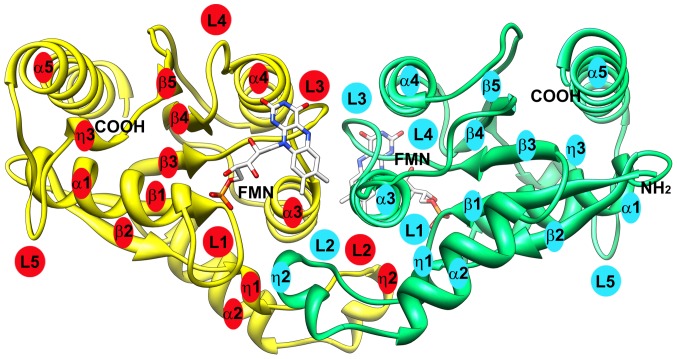
Overall structure of FerBHis_6_ from *P. denitrificans*. The two protein subunits are shown in yellow and green ribbon representation and two bound FMN cofactors as stick models. The secondary structural elements (five α-helices, three 3_10_ helices, five β-sheets and five loops) are designated α, η, β and L and numbered as they appear along the polypeptide chain.

The crystal structure of FerBHis_6_ in solution was determined by the multiple wavelength anomalous dispersion method using 1.40 Å resolution diffraction data (Table 1) from crystals of a selenomethionine derivative (PDB ID: 3U7R). The crystals belong to the orthorhombic space group P2_1_2_1_2 with two protein molecules in an asymmetric unit. The final model contains residues 2-182 (monomer A and B), one polyethylene glycol fragment, 451 water molecules and two flavin adenine mononucleotides. Most residues in the model are well defined in the 2*F_o_* - *F_c_* electron density map; missing density at the C terminus corresponds to the hexahistidine end (residues 184–190). The two monomers are identical with an r.m.s.d. (root mean square deviation) between equivalent C-α atoms of 0.08 Å. Each monomer has an α/β twisted open-sheet structure containing alpha helices on both sides of the central *β*-sheet in an arrangement typical for a flavodoxin-like fold. Five parallel strands in the order β2, β1, β3, β4, β5, are sandwiched between the helices α1 and α5 on one side and the helices α2, α3 and α4 on the other side. There are two 3_10_ helices (residues 40–42 and 48–53) between β2 and α2 and a short 3_10_ helix (residues 149–151) between β5 and α5 (Figure 1).

**Table 1:** Data collection and refinement statistics.

	3W-MAD data, Se-Met			Hi-res
Data set	Se-peak	Inflection point	High-energy remote	Se-Met
Space group	P2_1_2_1_2	P2_1_2_1_2	P2_1_2_1_2	P2_1_2_1_2
Unit-cell Parameters (Å)	*a* = 61.22	*a* = 61.25	*a* = 61.25	*a* = 61.03
	*b* = 89.19	*b* = 89.22	*b* = 89.24	*b* = 89.14
	*c* = 71.46	*c* = 71.49	*c* = 71.53	*c* = 71.28
Wavelength (Å)	0.97714	0.97753	0.97522	1.000036
Resolution range (Å)	30–1.75 (1.78–1.75)	30–1.75 (1.78–1.75)	30–1.75 (1.78–1.75)	19.11–1.40 (1.44–1.40)
*R*sym (%)	5.7 (33.7)	6.0 (41.2)	5.2 (41.7)	5.5 (28.0)
*I*/σ*I*	14.8 (2.2)	14.0 (1.8)	10.7 (1.4)	19.5 (3.3)
Completeness (%)	86.1 (68.0)	85.8 (66.0)	80.5 (57.7)	97.2 (86.2)
Redundancy	3.8 (1.9)	3.8 (1.9)	2.2 (1.3)	5.7 (2.2)
Wilson B factor (Å^2^)	21.8	22.9	23.7	15.0
**Refinement**				
Resolution (Å)				19.11–1.40 (1.44–1.40)
No. Reflections				72133 (4622)
*R*_work_/*R*_free_ (%)				16.0 (21.4)/17.4 (22.4)
No. Atoms				
Protein				2848
Ligand/ion				90
Water				451
B factor (Å^2^)				
Protein				9.368
Ligand/ion				11.465
Water				20.158
RMS deviations				
Bond lengths (Å)				0.013
Bond angles (°)				1.765
ESU (Å)				0.033

### Quaternary Structure of FerBHis_6_

The two molecules of the asymmetric unit form a head-to-tail homodimer with two symmetrical active sites facing the opposite sides and located at the interface between the subunits. Dimerization occurs mainly via anti-parallel packing of helices α3 and α4 into a four-helix bundle. A total of 23 residues from each monomer is involved in dimer formation (contact criteria Van der Waals overlap > −0.4 Å). The interface area of this particular assembly covers 1115.9 Å^2^ according to a surface area analysis by PISA [24], which strongly favors it against two other possible dimeric assemblies generated in the crystal lattice (interface areas 739.2 Å^2^ or 397.1 Å^2^). Moreover, the adjacent dimers might possibly interact through flat parts of their surfaces and pack perpendicular to one another to form a tetramer (buried area 12586.5 Å^2^).

In order to determine the actual oligomeric state of FerBHis_6_ in solution, size-exclusion chromatography was first carried out on a calibrated Superose 12 column. The protein eluted at a volume of 9.5 mL (Figure S1), corresponding to 1.88 subunits with molecular mass of 21 288 Da organized in a single molecule. The prevalence of a dimeric form of FerB at moderate dilutions agrees with the results of earlier studies using chemical crosslinking or light scattering [13]. The behavior in more concentrated solutions was assessed by SAXS. The theoretical SAXS profiles of the tetramer and dimer were calculated from the crystal structure by the CRYSOL program and compared with the experimental scattering curves obtained at protein concentration of 5, 2.5 and 1.25 mg/mL (Figure S2A). For concentrations 5 and 2.5 mg/ml, the experimental data were better fitted by the tetramer (χ_5_ = 1.69 and χ_2.5_ = 1.27) than the dimer (χ_5_ = 8.53 and χ_2.5_ = 5.02) model, while for the lowest, both fits gave nearly the same values χ_1.25_ = 1.22 for the tetramer and χ_1.25_ = 1.50 for the dimer. This difference indicates the occurrence of a dimer-tetramer equilibrium. Analysis of the same original data for all concentrations with the program OLIGOMER estimated the partial volume fraction of dimer:tetramer to be 9∶91% (χ^2^ = 1.18) at protein concentration 5 mg/mL, 13∶87% (χ^2^ = 1.18) at protein concentration 2.5 mg/mL and 34∶66% (χ^2^ = 1.60) at protein concentration 1.25 mg/mL (Figure S2B).

### Similarity to Other Proteins

**Figure 2:**
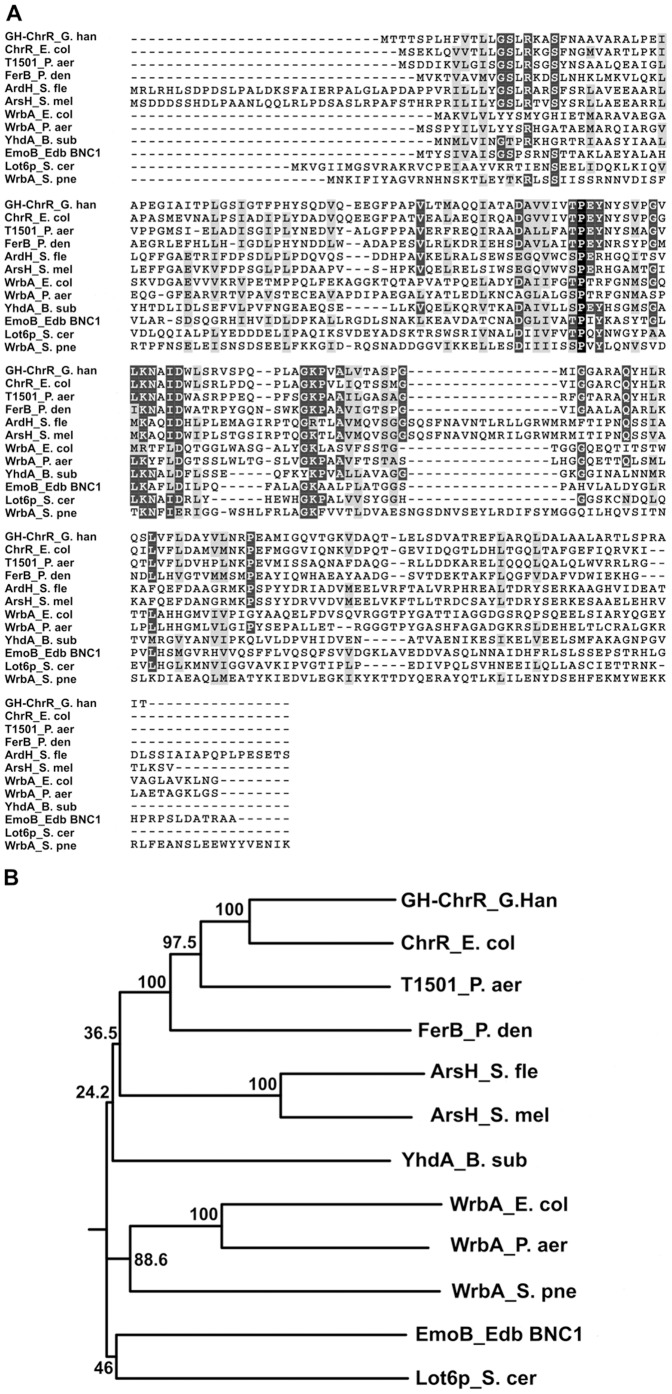
Tertiary structure-based sequence alignment (A) and phylogenetic tree (B) of selected structural homology proteins of FerB from a NADPH dependent FMN reductase family (PF03358). Strictly conserved residues are shown in black, dark gray shades indicate a high degree of conservation, while light grays indicate various degrees of low conservation. Generally, shaded residues indicate that a conservation of similar residues persists across at least 60% of the alignment. The alignment was generated using ClustalW with a BLOSUM62 matrix and default parameters and processed with BoxShade 3.31. Organisms and ORF designations from the corresponding genomic sequence: GH-ChrR_*G. hanseii* (D5QFC5, gi: 388603906), ChrR_*E. coli* (P0AGE6, gi: 84028020), T1501_*P. aeruginosa* (Q9I4D4, gi: 81541544), FerB_*P. denitrificans* (A1B9E3, gi: 69933457), ArsH_*S. flexneri* (Q7UC03, gi: 75386123), ArsH_*S. meliloti* (Q92R45, gi: 81634873), WrbA_*E. coli* (P0A8G6, gi: 67475535), WrbA_*P. aeruginosa* (Q9I509, gi: 81622522), YhdA_*B. subtilis* (O07529, gi: 81341002), EmoB_Edb (EDTA-degrading bacterium) BNC1 (Q9F9T2, gi: 75466236), Lot6p_*S. cerevisiae* (Q07923, gi: 74583672), and and WrbA_*S. pneumoniae* (Q97NR6, gi: 81531538). The phylogenetic tree with the greatest bootstrap values was constructed by a complete phylogenetic analysis package PHYLIP.

A search for structurally similar proteins using the structural database comparison program DALI returned about 9 hits with Z-scores above 15. The top 11 matches belonging to the protein family PF03358 and having at least 14% sequence identity with FerB are listed in Table S3. Amino acid sequences of these matches were aligned with ClustalW and PHYLIP was used to draw the phylogenetic tree (Figure 2). FerB clustered with two recently structurally characterized proteins, the *Escherichia coli* chromate reductase ChrR [25] and the *Gluconacetobacter hansenii* chromate reductase [26], and also with the *Pseudomonas aeruginosa* FMN reductase [27]. As it is shown in Figure 2A, all the 12 representatives included in the analysis share the similar conserved sequences thought to be involved in binding of flavin and NAD(P)H [27].

### Flavin Cofactor and its Binding Site

**Figure 3:**
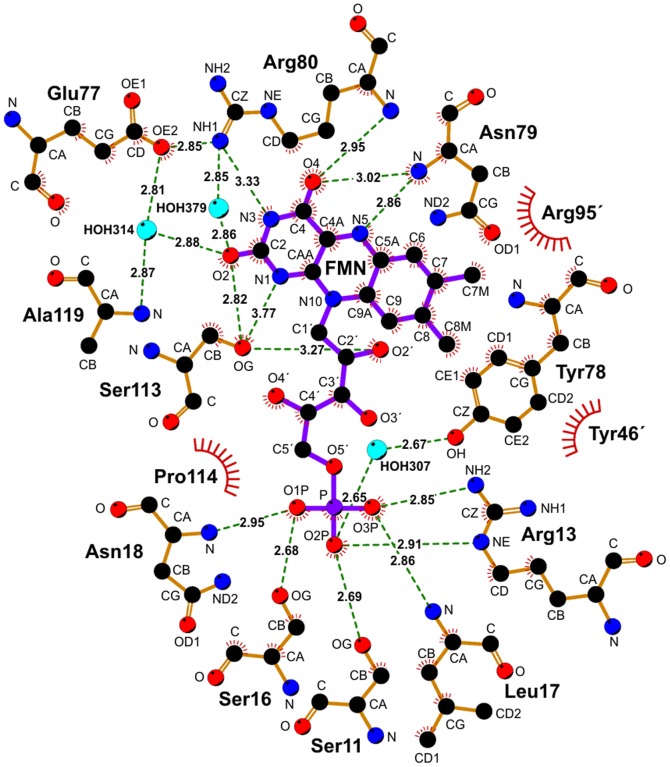
Predicted interactions between FMN and the protein. Hydrogen bonds are shown by dashed lines with the bond length (Å) printed in the middle, while the spoked arcs represent protein residues making nonbonded contacts with the ligand. The image was generated using the program LIGPLOT, version 4.5.3.

Contrary to the initial experimental hints [5], the present X-ray crystal data show unequivocally that FMN and not FAD is a true cofactor of FerB (Figure S3). The isoalloxazine ring of FMN in enzyme crystal is bent 12.8° along the N5-N10 axis. It is uncertain at present whether the initially planar oxidized isoalloxazine adopted this conformation as a result of binding to protein or whether it was only later converted to a bent semiquinone by photoelectrons generated during data collection [28]. The FMN binding site is mainly composed of residues from the loops L1 and L3, supplemented by a few residues belonging to the adjacent subunit (Tyr46, Asp48, Arg95). The proposed interactions are summarized schematically in Figure 3. L1 carries a variant of the (T/S)*X*R*XX*S*X*(T/S) motif for phosphate binding [27], namely SLRKDSLN, of which Ser11, Arg13, Ser16, Leu17, and Asn18 can form hydrogen bonds with the nonbridging oxygen atoms of the FMN phosphate. The amino acid residues 77 to 80 of L3 are expected to participate as follows. Glu77 and Arg80 are bonded with one another and maintain two molecules water in positions which allow to protonate the O2 atom of isoalloxazine. Alternatively, the proton for O2 can come from the hydroxyl group of Ser113. Tyr78 probably binds FMN through a stacking interaction with the dimethylbenzene end of the isoalloxazine ring; the phenolic hydroxyl group of Tyr78 could have a hydrogen bond with a phosphate oxygen atom via a molecule of water. The peptide backbone nitrogen atom of Asn79 and Arg80 can interact with N5 and O4 of isoalloxazine. As a result, FMN is anchored into the pocket in such a way that the pyrimidine end on the isoalloxazine ring remains accessible to the solvent and thus available for interacting with electron donors and acceptors.

### Preparation and Characterization of Apoenzyme

The immobilized metal-affinity chromatography method of apoflavoprotein preparation, originally developed for the flavin-containing PAS domain of the NifL protein from *Azotobacter vinelandii* [29], proved to be convenient also for FerBHis_6_, although the concentrations of urea and KBr had to be raised to 4 M for both in order to remove FMN most effectively. The procedure yielded apoenzyme with less than 0.1% residual enzymatic activity and no appreciable absorption in the visible region. At least 99% of the original activity could be recovered upon incubation with a surplus of the native flavin. Elution volume in the gel permeation chromatography (Figure S1) of apoFerBHis_6_ were similar if not identical to that of FerBHis_6_, demonstrating that the apoenzyme lacking FMN still retains the ability to form a homodimer. The deflavinated product showed decreased storage stability and precipitated slowly after being transferred to low ionic strength buffers. When subjected to differential scanning calorimetry, it gave an irreversible endothermic peak at 56.5 °C, which contrasts with a significantly higher temperature of 81.2 °C for denaturation of the FMN-containing holoenzyme (Figure S4). Thus, besides its catalytic role, the flavin cofactor also displays a structural function by stabilizing the dimeric FerB protein.

### Binding Affinity of Apoenzyme for Flavin

**Figure 4:**
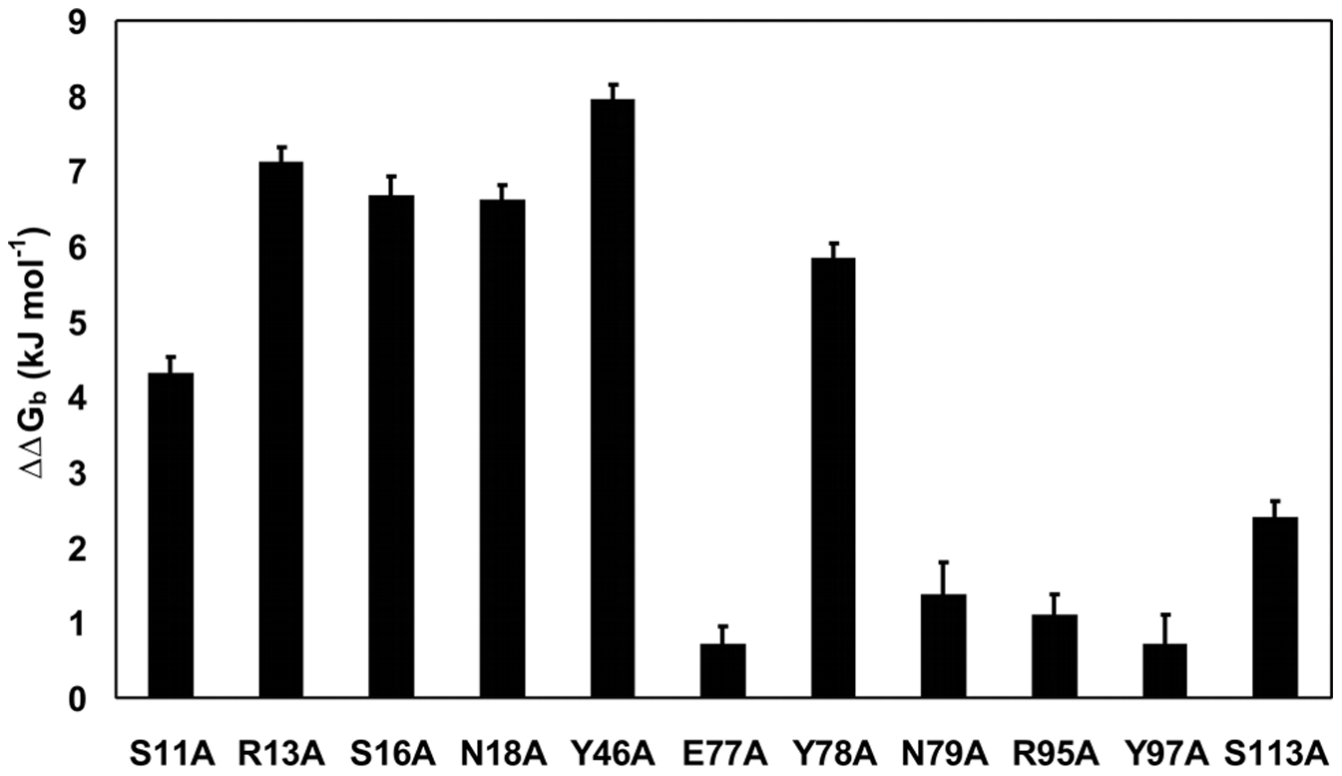
Changes in the standard Gibbs energy for FMN binding to alanine substitution mutants of FerBHis_6_. The depicted values of ΔΔ*G_b_* were calculated from the dissociation constants for the mutant and wild-type protein as determined by fluorimetric titration (see Materials and Methods).

Consistently with the behavior of many other flavoproteins, binding of free FMN to apoFerBHis_6_ and its mutant forms was accompanied by fluorescence quenching, which provided a means to determine the ligand dissociation constants (*K_d_*). The *K_d_* values for complexes of apoFerBHis_6_ with FMN and with riboflavin, which lacks the 5′-phosphate group of FMN, were found to be 27±2 nM and 6.58±0.05 µM respectively (25 mM Tris-HCl, pH 7.4, 25 °C). A 244-fold increase in *K_d_* for riboflavin indicated that interaction of apoenzyme with the FMN phosphate significantly stabilizes the complex. This conclusion could be further substantiated through mutating amino acid residues, located near the phosphate terminus of the bound FMN, to alanine. The changes in the Gibbs free energy of binding (ΔΔ*G_b_*) upon mutation were significant for Ser11, Arg13, Ser16 and Asn18 (Figure 4), giving quantitative support to the idea that these amino acid residues constitute the binding site for phosphate. Similar mutational analysis demonstrated the importance of the tyrosine residues Tyr46 and Tyr78 and possibly also Ser113 for capturing the isoalloxazine ring of FMN.

### Effect of Mutations on Kinetic Parameters for Oxidation of NADH

FerB is known to catalyze oxidation of NAD(P)H by a variety of electron acceptors. To identify residues involved in NADH recognition and catalytic activity, we determined kinetic parameters of the mutant holoenzymes in the presence of a fixed concentration of UQ-0 (0.1 mM) and variable concentrations of NADH. Mutation to alanine of Ser11, Arg13, Ser16, Asn18, Tyr46, Tyr78 and Asn79 did not impair the reaction at all, whereas mutations at residues Glu77, Arg80, Arg95, Ser113 and Gly115 were effective in decreasing *k_cat_* and for most cases in increasing *K_m_* (Table 2). The Glu77-Arg80 pair located near the pyrimidine side of the isoalloxazine ring of FMN proved to be fundamentally important for enzyme functioning, since elimination of either member by charge-reversal or charge-neutralizing mutation (Glu77 to Lys, Leu or Met or Arg80 to Glu, Leu or Met) reduced *k_cat_* by 2 to 4 orders of magnitude. The observed changes in the apparent *K_m_* values imply that NADH binding was mostly affected when mutations occurred at positions Arg80 and Gly115.

**Table 2:** Kinetic parameters and standard redox potentials of FerBHis_6_ for the wild-type and mutant variants.

	*K_m_* (μM)	*k_cat_*(s^−1^)	*k_cat_*/*K_m_* (mM^−1^ s^−1^)	*E_m_* (mV)	*k_obs_* (s^−1^)
FerBHis_6_	22±2	258±6	11727±1100	−138±2	147.01±0.35
FerBHis_6_-E77A	180±30	17.9±3.0	99±23	−225±10	
FerBHis_6_-E77K	30±8	0.75 ± 0.05	25±7	−239±7	0.228±0.001
FerBHis_6_-E77L	250±30	0.072±0.006	0.29±0.04	−229±8	
FerBHis_6_-E77M	420±160	2.5±1.0	6.0±3.3	−223±4	
FerBHis_6_-Y78A	40±10	196±13	4900±1267		
FerBHis_6_-R80E	30±3	0.275 ± 0.006	9.2±0.9	−230±7	0.608±0.003
FerBHis_6_-R80K	350±80	9.5±1.4	27±7	−148 ± 2	
FerBHis_6_-R80M	780±280	0.302±0.084	0.39±0.18	−148±6	
FerBHis_6_-R80L	580±150	0.142 ± 0.030	0.24±0.08		
FerBHis_6_-R95A	20±2	146±4	7292±756	−217±11	
FerBHis_6_-R95E	70 ± 9	21.1±0.9	302±41	−245±1	45.15±0.09
FerBHis_6_-S113A	60±20	76.7±7.2	1278±443	−239±1	27.88±0.03
FerBHis_6_-G115F	410±90	2.2±0.3	5.4±1.4		2.607±0.007
FerBHis_6_-G115I	630±70	1.7±0.1	2.7±0.3	−149±3	
FerBHis_6_-G118F	85 ± 6	77±9	906±124		
FerBHis_6_-G118I	38±3	12±1	316±36		

The apparent Michaelis constant (*K_m_*) for NADH and the catalytic constant (*k*_cat_) were obtained by the initial velocity kinetic analysis at a constant UQ-0 concentration of 0.1 mM. The standard two-electron redox potential (*E° ´*) was determined spectrophotometrically by equilibrating the protein with a redox dye. The first-order rate constant for flavin cofactor reduction (*k_obs_*) was measured upon rapid mixing the enzyme with 5 mM NADH at 10 °C under anoxic conditions. Experimental details on these measurements are given under Experimental Procedures section.

### Flavin Cofactor Reducibility

**Figure 5:**
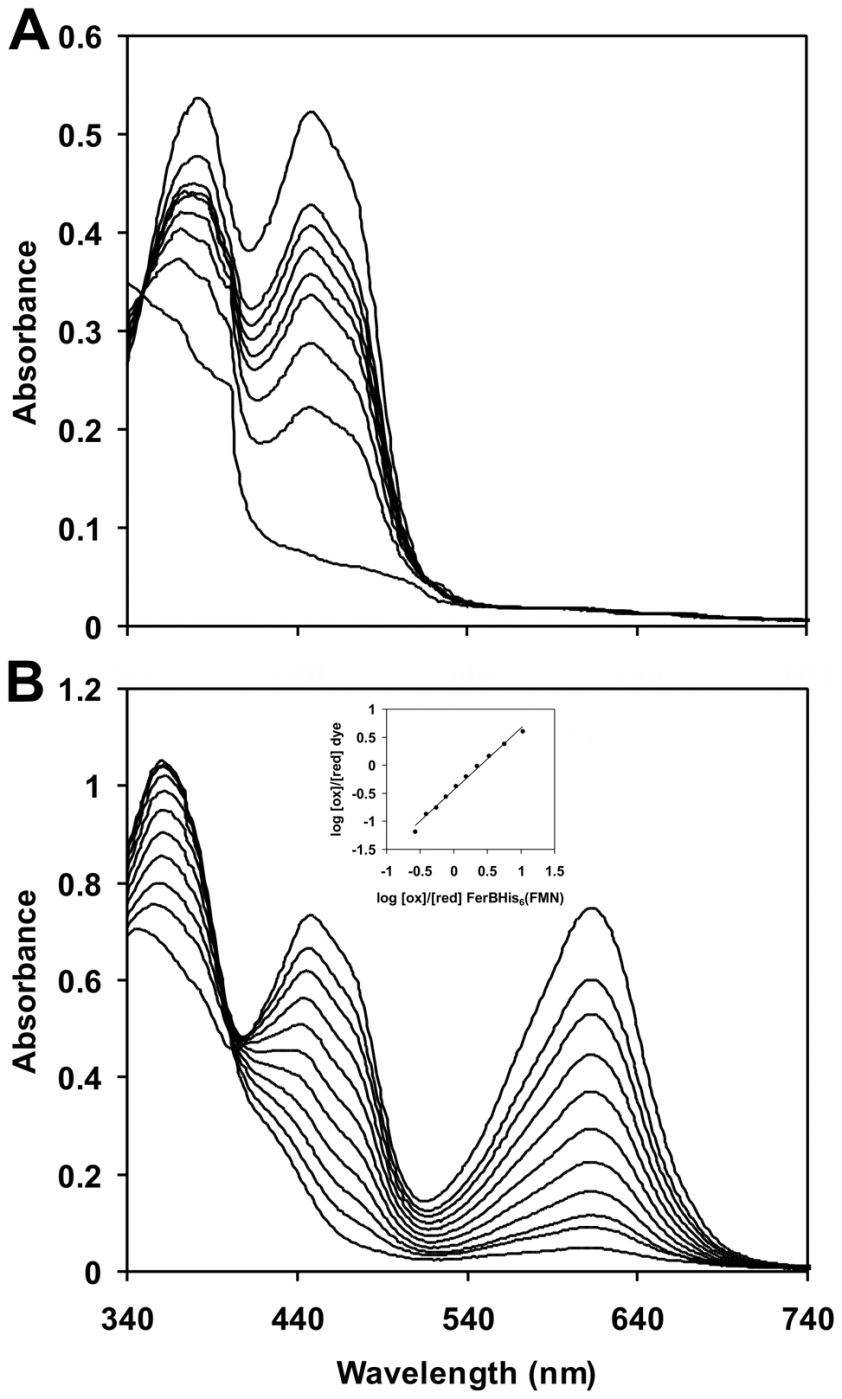
Spectral changes during the anaerobic reduction of FerBHis_6_ by the xanthine/xanthine oxidase system. The spectra shown (from top to bottom) were recorded at 0, 5, 10, 13, 17, 22, 26, 30 and 36 min for 48 µM FerBHis_6_ (A) and 0, 5, 10, 15, 20, 24, 27, 30, 33, 37 and 40 min for 65 µM FerBHis_6_ with 40 µM Indigo Carmine. In (B), the inset shows the log(ox/red) for the dye vs. that for the enzyme bound FMN revealing a slope of 1.08, which is close to the theoretical value of 1. The standard potential of FMN was calculated from the *y*-axis intercept.

Upon anaerobic exposure to xanthine/xanthine oxidase system at pH 7.0 the yellow flavin chromophore of FerBHis_6_ was gradually reduced to its hydroquinone form without any noticeable formation of a blue or red semiquinone radical (Figure 5A). The inclusion of indigo carmine (*E° ´* = −125 mV, [30]) enabled us to determine the standard redox potential difference between the flavin cofactor and the reference dye (Δ*E° ´*). If a true redox equilibrium is attained, a plot of the log([ox]/[red]) ratio of the enzyme versus the corresponding log([ox]/[red]) ratio of the dye should produce a straight line having a slope of *n_F_*/*n_D_* and an intercept on the vertical axis of –*n_F_*Δ*E° ´*/59, where *n* denotes the number of electrons needed for complete reduction of flavin (F) or dye (D). Plotting the data in this way (Figure 5B, inset) gave a slope of 1, which is close to unity as expected for *n_F_* = *n_D_* = 2. From the intercept value of −0.45 the *E° ´* of bound FMN could be calculated as −125 + ((59/2) × −0.45) = −138 mV. The experiment of Figure 5 was repeated with mutant enzymes and, when appropriate, with the alternative reference dyes phenosafranin (*E° ´* = −252 mV, [30]) or anthraquinone-2-sulfonate (*E° ´* = −225 mV, [31]). The results are listed in the penultimate column of Table 2. Testing our system with 50 µM free FMN, we determined an *E° ´* of −212 mV which is close to the published value of −207 mV [32]. The positive shift in *E° ´* for FerBHis_6_ means that the wild-type apoenzyme binds the oxidized flavin more weakly than the reduced form, which facilitates the cofactor reduction. This preference is lost in all of the tested mutants at Glu77, in the S113A mutant and also in the R80E and R95E mutants where the positively charged guanidinium group of arginine is replaced by the negative carboxyl group of glutamate.

The reduction of the FMN group by NADH, a plausible physiological donor, was evaluated spectrophotometrically in a stopped-flow apparatus under anaerobic conditions and at 10 °C. Changes in flavin absorbance at 450 nm upon rapid mixing of 30 µM FerBHis_6_ with 1–5000 µM NADH (final concentrations) followed single exponential decay kinetics. The fitted value of *k_obs_* responded hyperbolically to the NADH concentration with the half maximal effect at 6.4±1.0 µM and tended toward a limit of 147.0±0.4 s^−1^ that can be considered as an apparent electron-transfer rate constant for the FerB-NADH complex. The *k_obs_* determined at saturating concentration of NADH (5 mM) did not depend on pH in the interval 3–9. By substituting 99% deuterium oxide for normal water in buffer of pH 6.8, a kinetic solvent isotope effect *k_obs_*(H_2_O)/*k_obs_*(D_2_O) of 1.9 was noted. The *k_obs_* at 5 mM NADH for the mutant proteins were found to be diminished in cases where the enzymatic reaction of NADH with quinone was also impaired by the mutation (Table 2). Attempts were also made to measure the oxidation of the flavin cofactor, pre-reduced with an equivalent of sodium dithionite, by 200 µM UQ-0, but the reaction of all proteins was too rapid and completed within the dead time of the stopped-flow instrument (0.4 ms).

### Stereochemistry of Flavin Cofactor Reduction

Knowledge of the crystal structure of the FerB holoenzyme was enough to predict hydrogen transfer to and from the *si*-face of the bound FMN (Figure 1). However, because our attempts to grow crystals of FerB in complex with NADH or NAD^+^ had failed, we were still left uncertain as to the stereospecificity of hydrogen removal from C4 of the dihydronicotinamide moiety of NADH. Therefore, we examined the stereochemical outcome of the FerB-mediated oxidation of NADH that was stereospecifically labeled with deuterium at either the *pro-R* or *pro-S* position. Only in the former case the ^1^H NMR spectrum of the NAD^+^ product contained a signal at 8.8 p.p.m (marked by arrow in Figure S5A) typical for the 4-position of nicotinamide, so we could conclude that it is the *pro-S* hydrogen that is transferred to FMN. Quantification of the kinetic isotope effects (KIEs) for the enzyme-catalyzed UQ-0 reduction (NADH, 0.15 mM; UQ-0, 100 µM; FerBHis_6_, 0.018 µM; pH, 7.4; 30 °C) and for the enzyme-bound flavin cofactor reduction (NADH, 1 mM; FerBHis_6_, 50 µM; pH, 7.4; 10 °C) using the C4 monodeuterated enantiomers of NADH gave the average KIE ratios of 5.45±0.21 and 6.15±0.34 for [4S-^2^H]NADH and 1.07±0.13 and 1.05±0.05 for [4R-^2^H]NADH. The lower reactivity of the deuterated *S*-enantiomer can be taken as confirmation of the proposed stereochemistry.

### FMN and NADH Docking

The laboratory-based studies on interaction of the FerB protein with FMN and NADH were complemented with an *in silico* analysis using the *AutoDock Vina* suite. Initially, in order to assess the reliability of the docking method, FMN was picked up from the holoenzyme atomic structure (PDB ID: 3U7R) and then idealized coordinates of FMN was docked back into the rigidly fixed binding site. As illustrated in Figure S6, the docked conformation of FMN with the lowest binding energy closely resembles its crystal structure, with r.m.s.d. value of 1.1 Å for 31 non-hydrogen FMN atom pairs. The calculated *K_d_* value of 9.9 nM agrees approximately with that obtained experimentally (27 nM, see above). NADH was docked into to the crystal structure of holoenzyme where reduced form of FMN was replaced by its oxidized form to resemble the proton transfer reaction. The complex with the lowest binding energy that matches the experimentally determined stereochemistry was chosen from approximately 100 docking results. In the putative complex (Figure S7) the nicotinamide ring of NADH is stacked over the isoalloxazine ring of FMN with a central distance of 4.6 Å and an angle of their ring planes of 30.0°. The distance of 3.8 Å between the C4 of NADH and N5 of FMN in this complex allows the hydride transfer to occur. The residues Arg80 and Arg95 were predicted to contact the nicotinamide moiety. The carboxyamide oxygen of the NADH (O7N) interacts with Arg95 (3.0 Å), while the oxygen of the ribose (O2D) interacts with Arg80 (2.9 Å). The diphosphate moiety of the NADH could interact via its O2A with the amide N of Gly115 (3.8 Å), while the adeninosine moiety interacts via O3B with Arg95 (3.2 Å) and via N6A and N7A with Arg13 (3.4 Å resp. 3.3 Å). The calculated *K_d_* for the holoenzyme-NADH complex (4.4 µM) is consistent with the experimental *K_m_* value for NADH (Table 2).

### Engineering of the Flavin Reductase Activity

**Figure 6:**
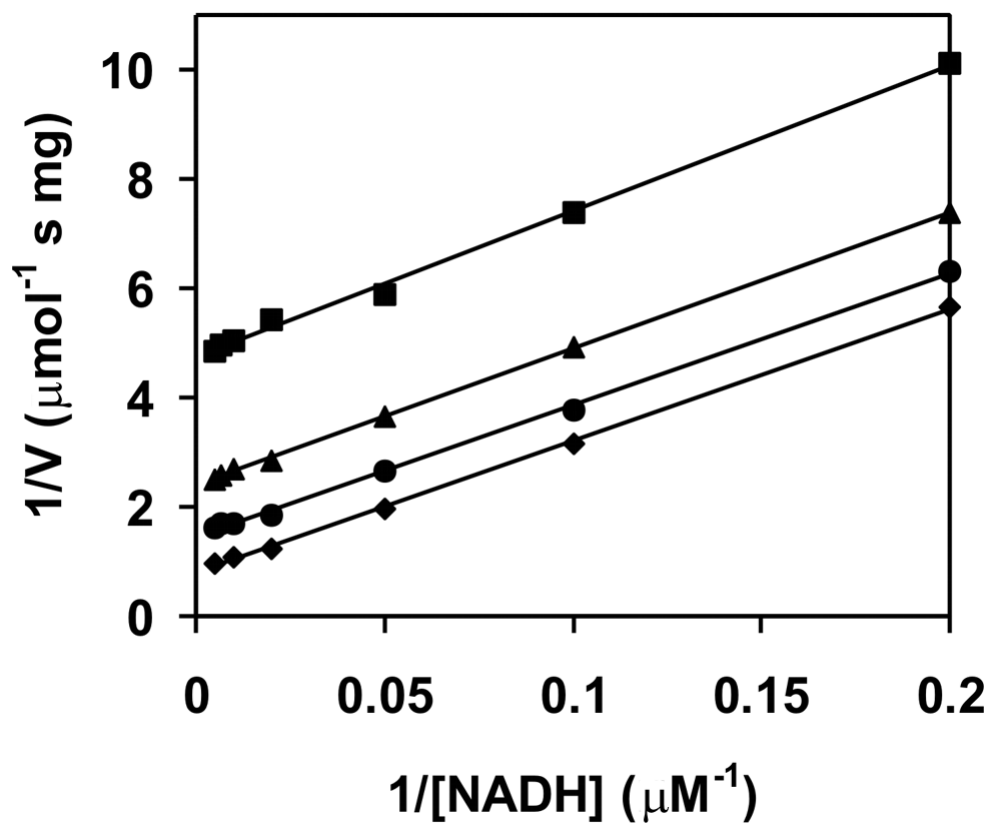
Initial-velocity kinetics of the NADH:FMN oxidoreductase reaction catalyzed by FerBHis_6_-R95A. Measurements were performed at 30 °C with 61 nM enzyme in 25 mM Tris-HCl buffer (pH 7.4). Reciprocal initial velocity is plotted against the reciprocals of NADH concentration at a series of fixed concentrations of FMN equal to 10 (squares), 20 (triangles), 50 (circles) and 100 (rhombs) μM.

Although FerB belongs to the family of NADPH-dependent FMN reductases based on sequence homology, in fact it shows very little activity toward external FMN as compared to that toward UQ-0 as a reference, the ratio of the respective *k_cat_* values being only about 0.006. A possible reason may be a steric hindrance preventing the binding of two flavins at once. Inspection of the structure suggested that removal of the bulky arginine side chain from position 95 might enlarge the active site pocket so that the accommodation of the second flavin molecule could be facilitated. In line with this expectation, we found that the *k_cat_* ratio increased to 0.32 as a result of the R95A mutation. The flavin reductase activity of the novel mutant enzyme was subjected to a bisubstrate kinetic analysis (Figure 6). A double reciprocal plot of initial velocities versus concentrations of NADH as the varying substrate at four fixed levels of FMN gave a set of parallel lines indicating a ping-pong type of kinetic mechanism. By fitting the data by non-linear regression to the appropriate kinetic model, the catalytic constant and the Michaelis constants for NADH a FMN were calculated to be 50±5 s^−1^, 33±7 µM and 111±15 µM. Riboflavin and FAD were also substrates exhibiting *k_cat_* values of 75±10 s^−1^ and 38±1 s^−1^ and *K_m_* values of 59±12 µM and 95±7 µM.

Since the side chain of Arg95 protrudes into active site from the neighbor subunit across the dimeric interface, more extensive changes in quaternary structure had to be considered as an alternative explanation for the effect of the mutation. However, the persistence of a dimeric state of the mutant proteins R95A and R95E, as verified by gel permeation chromatography (Figure S1) and SAXS (data not shown), renders this possibility unlikely.

## Discussion

Proteins of the flavodoxin type display a large range of FMN binding affinity. The apoproteins of typical flavodoxins bind FMN with a subnanomolar or even picomolar *K_d_* [33], whereas the WrbA protein of *E. coli* has a *K_d_* as high as 2 µM [34]. FerB (*K_d_* = 22 nM) thus represents an in-between case. The much weaker binding of FMN to WrbA compared with that seen for flavodoxins was attributed to alterations of critical residues located near the plane of the isoalloxazine ring of FMN and to the reduced number of hydrogen bonds with the phosphate group [35]. For the apoflavodoxin from *Anabaena* PCC 7119, pivotal binding studies by Lostao et al. [22] exploiting not only FMN but also its shortened analogues riboflavin and lumiflavin led to a dissection of the overall Gibbs binding energy of FMN (−55 kJ mol^−1^) into three components related to the phosphate (−29 kJ mol^−1^), isoalloxazine (−21 kJ mol^−1^), and ribityl (−5 kJ mol^−1^) parts of the molecule. An analogous calculation with the experimental *K_d_* values for FMN (22 nM) and riboflavin (6.6 µM) shows that only −13.6 kJ mol^−1^ of the total −43.2 kJ mol^−1^ apparently comes from the phosphate, i.e., that the relative importance of isoalloxazine may be higher for FerB than for the *Anabaena* flavodoxin. In most flavodoxins isoalloxazine is sandwiched between the aromatic residues tyrosine and tryptophan at the *si*-face and *re*-face, respectively [36]. In FerB only the *re*-face residue remains aromatic (Tyr78) and, as indicated by mutant analysis, adds to the binding, the contribution of the *si*-face arginine (Arg95) being negligible. Yet another tyrosine residue (Tyr46), which extends from the second subunit and has its phenolic ring positioned nearly perpendicularly, appears to interact with the flavin at its dimethylbenzene end by providing a hydrophobic environment. The binding of the flavin ring is further strengthened by hydrogen bonds with Ser113 and two backbone NH groups (not quantifiable by alanine mutagenesis). Alanine substitutions were also helpful in confirming the presumed role of Ser11, Arg13, Ser16 and Asn18 in binding the phosphate moiety. The sum of ΔΔ*G_b_* values of the individual mutations equals 24.7 kJ mol^−1^, which greatly exceeds the value of 13.6 kJ mol^−1^ expected from the FMN and riboflavin binding data. We interpret this discrepancy to mean that the ΔΔ*G_b_* values cannot be simply added because there are some cooperative interactions among the spatially close residues.

Detailed structural studies of NADH binding to FerB-like enzymes are generally hampered due to the lack of co-crystals suitable for X-ray diffraction. At present, the only available data are those of the NADH:FMN oxidoreductase (EmoB) from *Mesorhizobium* sp. BNC1 [37] that has 21% sequence identity with FerB. In the crystal structure of the EmoB-FMN, NADH complex (PDB ID: 2VZJ), the *si*-face (B-side) of the nicotinamide ring of the pyridine nucleotide packs against the *si*-face of the isoalloxazine ring of FMN, as is likely to be the case for FerB. The amine group of Lys81 and the backbone carbonyl group of Gly112 in EmoB were considered to bind to the carboxamide and phosphate groups of NADH, respectively [37]. According to the results of amino acid sequence comparison (Figure 2A), the amino acid at position 81 is not conserved and EmoB is the only member that has a lysine at this site. The equivalent residue in FerB, Asn79, was mutated to Ala without affecting enzyme activity. On the contrary, Gly112 of EmoB is strictly conserved among all the sequences. The corresponding Gly115 in FerB is located on a turn between strand β4 and helix α4 within a sequence motif SXGXXG that is reminiscent of the common GXGXXG fingerprint region of the classical Rossmann fold. These glycine-rich regions are typically involved in positioning dinucleotides in a correct conformation by allowing for close contact of the polypeptide chain with its central diphosphate portion [38]. Our finding that introduction of a large phenylalanine or isoleucine substitution at amino acid 115 results in a one-order increase in *K_m_* for NADH and two-order decrease in *k_cat_* (Table 2) is consistent with (but does not in itself prove) the idea of Gly115 being a part of the NADH binding site. In our model of the FerB-NADH docked complex, Gly115 lies indeed within hydrogen-binding distance of a O2-phosphate oxygen of NADH and clear steric hindrances occurs following superimposition of Phe or Ile on it. The same Phe or Ile substitutions at the more distant Gly-118 were much less effective. Arg80 may also participate in NADH binding, since its substitutions strongly affected the *K_m_* value. In this case the docking model predicts interaction with the hydroxyl group of NADH ribose.

New structural data link FerB very closely to the recently characterized chromate reductases (ChrR) of *Escherichia coli* (PDB ID: 3SVL) and *Gluconacetobacter hansenii* (PDB ID: 3S2Y). One thus would expect that all three enzymes might operate by the same overall mechanism, but this need not necessarily be true. Previously it was claimed that the reduction of chromate by NADH catalyzed by *G. hansenii* ChrR proceeds via an ordered, sequential reaction with chromate binding first followed by NADH and that binding of NADH alone produces a death-end complex [26]. Some facts about FerB, however, contradict this picture. When catalyzing a reaction of between NADH and quinone, FerB obeys a ping-pong type reaction mechanism [5], which is consistent with a temporary parking of reducing equivalents from NADH at the enzyme-bound flavin cofactor. Prestationary assays reported here show that the flavin group of FerB is still reducible by NADH despite the absence of any electron acceptor like quinone or chromate. The estimated limiting *k_obs_* (∼140 s^−1^ at 10 °C) is similar to the steady-state value of *k_cat_* for the NADH-driven reduction of UQ-0. Taking also into account a comparable deuterium kinetic isotope effect upon both processes, we conclude that (i) an electron-acceptor-independent reduction of flavin is operative, (ii) it is kinetically competent to account for the observed enzyme activity, and (iii) it may be rate-limiting for the whole catalytic cycle, at least with UQ-0 as electron acceptor.

Following hydride transfer to the N5 position of the flavin, stabilization of the negative charge evolving at the N1-C2 = O2 locus of the isoalloxazine moiety is required. In flavoenzymes this is usually accomplished through the interaction with a positively charged entity, either fully charged, such as a Lys or Arg side chain, or partially charged, such as the N terminus of a helix or a cluster of peptide nitrogen [39]. In the canonical crystal structure of the rat liver quinone reductase (PDB ID: 1QRD) O2 of the FAD cofactor is hydrogen-bound to the backbone amide groups of Gly149 and Gly150 and also to the side chain of Tyr155. It was postulated that Tyr155 and the neighboring His161 form a charge-relay system needed for the reaction to take place without unfavorable charge separation [40]. FerB and the ChrR proteins of *E. coli* and *G. hansenii* are distinguished by a different microenvironment of the N1-C2 = O2 locus, comprising a hydroxyl group of serine and a water molecule hold in position by a glutamate-arginine pair. The residues Ser113 and Glu77 of FerB are well conserved among the homologous sequences (Figure 2A).The importance of Ser113 gains support from experiments of the alanine mutant that exhibits lower specific activity as well as depressed FMN binding. The involvement of water in catalysis by FerB is consistent with the observed solvent isotope effect of 1.9 for FMN reduction, suggesting that an oxygen-hydrogen bond is broken in a rate limiting step. Water has already been proposed to serve as a proton donor to N1 or O2 atoms at flavin reduction in several other flavoenzymes, such as NADPH-cytochrome P450 oxidoreductase (PDB ID: 1AMO; [41]), 2,4-dienoyl-CoA reductase (PDB ID: 1PS9; [42]), FMN-dependent azoreductase (PDB ID: 2HPV; [43]) and peptidyl-cysteine decarboxylase (PDB ID: 1G5Q; [44]). A crucial role of Glu77, amply substantiated by the results of mutational experiments, probably relates to maintaining the architecture of the active site. Unlike FerB, where a pair Glu77-Arg80 exists, the homologous glutamate in both of the ChrR enzymes pairs with a more remote arginine located 43 amino acid residues away. In *E. coli* ChrR this arginine lies at the surface and participates in dimer-dimer interaction [25]. Subtle differences in hydrogen bond networks in ChrR and FerB thus may explain why the former enzyme forms a stable homotetramer while the latter remains dimeric in dilute solutions.

Of the proteins similar to FerB listed in Table S3, five have been demonstrated to catalytically reduce flavin, i.e., to act as an NADH:flavin oxidoreductase. Judging from their crystal structures, these enzymes have the flavin cofactor largely exposed to the bulk solvent, whereas in FerB the dimethylbenzene part of the flavin nucleus appears to be shielded by the side chain of the Arg95 residue from the neighbor protomer within the homodimeric structure. The mutant protein R95A, prepared from FerBHis_6_ to relieve the presumed steric constraint, indeed gained the ability to reduce FMN with a *k_cat_* of 50 s^−1^, which is close to the value for the *B. subtilis* YhdA (60–70 s^−1^, [45]) but lower than that for EmoB of *Mesorhizobium* sp. bacterium BNC1 (397 s^−1^, calculated from the V_max_ of 1111 µmol min^−1^ mg^−1^ given in [37], using a subunit molecular mass of 21 412 Da). Similarly to EmoB, the engineered FerB obeyed a ping-pong kinetic mechanism with NADH and FMN as substrates (Figure 6) suggesting that NADH and a loosely associated FMN molecule interact in an alternating fashion with the same pocket containing another, tightly bound FMN. Such a mechanism was previously identified for EmoB through preparation and structural analysis of its FMN-NADH and FMN-FMN complexes [37]. Since a single mutation increasing the accessibility of the bound FMN sufficed for FerB to become an active flavin reductase with affinities for FMN, FAD and riboflavin (indicated by *K_m_* values) comparable each other, isoalloxazine rings contacts may be of importance in recognition of the external flavins. According to the ^1^H NMR chemical shift measurements, FMN self-associates in aqueous solutions with an association constant in the range 20–100 M^−1^ [46]. This would give an expected dissociation constant of the order of 10 mM, which is however still two orders of magnitude above the estimated Michaelis constants (∼0.1 mM). Pre-existing protein environment around the bound flavin cofactor thus probably also contributes to the external flavin substrate binding. A close relationship of FerB to the flavin reductases, demonstrated now both structurally and biochemically, may shed some light on the evolution of flavin reduction and various promiscuous catalytic activities of flavoproteins in general.

## Supporting Information

Table S1 The mutagenic oligonucleotide primers for site-directed mutagenesis of FerB His_6_.
(DOC)

Table S2 Data collection from SAXS.
(DOC)

Table S3 Amino-acid identity and structural similarity across the structural homologs of FerB in a FMN reductase protein family (PF03358).
(DOCX)

Figure S1 Superose 12 analytical size exclusion chromatography of FerBHis_6_ holoenzyme (1), apoenzyme (2), and FerBHis_6_-R95E mutant (3). The calibration curve used to estimate the native molecular weight based on the elution position is indicated.
(TIF)

Figure S2 **SAXS scattering curves.** X-ray scattering of FerBHis_6_ was compared with calculated scattering of the dimer (*dashed line*) and the tetramer (*solid line*) computed by CRYSOL for concentrations 1.25 (A), 2.5 (B) and 5.0 (C) mg/mL. The program OLIGOMER was used to estimate dimer and tetramer volume fractions for concentrations 1.25 (D), 2.5 (E) and 5.0 (F) mg/ml.
(TIF)

Figure S3 **Electron density map of FMN.** Stereo view of the 2*F_0_* – *F_c_* electron density map contoured at 1.0 *σ* cut-off corresponding to FMN at the active site.
(TIF)

Figure S4 **Thermal stability of apoFerBHis_6_ (A) and FerBHis_6_ (B) followed by DSC.** Protein concentrations were adjusted to 4.3 mg/mL for apoFerBHis_6_ and 2.5 mg/mL for FerBHis_6_ in buffer C and data were collected from 25 to 95 °C at a heating rate of 60 °C per hour.
(TIF)

Figure S5 **NMR spectra of NAD^+^.** The ^1^H NMR aromatic region of NAD^+^ formed from [4B-^2^H]NADH (A) or [4A-^2^H]NADH by FerBHis_6_ in the presence of 1,4-benzoquinone showing a resonance signal at 8.8-8.9 p.p.m. (A) or no resonance signal (B). The enzyme removed the proton from B-side (pro-*S*, *si*-face) of the dihydrogennicotinamide ring.
(TIF)

Figure S6 Superimposition of FMN from the crystal structure of FerBHis_6_ (white) and from the docking-derived complex (yellow).
(TIF)

Figure S7 **Structure of the docked FerBHis_6_-NADH complex.** NADH was modeled into the FerBHis_6_ structure using *AutoDock Vina v1.1.2*. FMN and NADH are shown with yellow and white carbons, respectively (A). The nicotinamide ring was stacked on top of the isoalloxazine ring at distance 3.8 Å that corresponding with distances of other NADH-dependent FMN reductases. The residues are depicted in green and violet to represent belonging to two different subunits. The known crystal structure of EmoB complex with NADH (PDB ID: 2VZJ) is included for sake of comparison (B).
(TIF)
