# Supplementary material for: The Structural and Functional Basis of Catalysis Mediated by NAD(P)H:acceptor Oxidoreductase (FerB) of Paracoccus denitrificans
Source: PLoS One. 2014 May 9;9(5):e96262. doi: 10.1371/journal.pone.0096262 (PMC4015959; doi:10.1371/journal.pone.0096262)
Supplement: Table S1 — The mutagenic oligonucleotide primers for site-directed mutagenesis of FerB His6. (DOC) [file pone.0096262.s001.doc]

| Primers | Sequence ^*^ |
| --- | --- |
| S11A-FP | 5´-CCG TCA TGG TGG GAG CCC TCC GCA AGG ATT C-3´ |
| S11A-RP | 5´-GAA TCC TTG CGG AGG GCT CCC ACC ATG ACG G-3´ |
| R13A-FP | 5´-CAT GGT GGG ATC GCT CGC CAA GGA TTC GCT CAA T-3´ |
| R13A-RP | 5´-ATT GAG CGA ATC CTT GGC GAG CGA TCC CAC CAT G-3´ |
| R13Q-FP | 5´-CAT GGT GGG ATC GCT CCA GAA GGA TTC GCT CAA TCA-3´ |
| R13Q-RP | 5´-TGA TTG AGC GAA TCC TTC TGG AGC GAT CCC ACC ATG-3´ |
| S16A-FP | 5´-GAT CGC TCC GCA AGG ATG CCC TCA ATC ACA AGC TGA-3´ |
| S16A-RP | 5´-TCA GCT TGT GAT TGA GGG CAT CCT TGC GGA GCG ATC-3´ |
| N18A-FP | 5´-CGC TCC GCA AGG ATT CGC TCG CCC ACA AGC TGA TGA AGG TAT T-3´ |
| N18A-RP | 5´- AAT ACC TTC ATC AGC TTG TGG GCG AGC GAA TCC TTG CGG AGC G-3´ |
| S11A/R13A-FP | 5´-CCG TCA TGG TGG GAG CGC TCG CCA AGG ATT CGC TCA-3´ |
| S11A/R13A-RP | 5´-TGA GCG AAT CCT TGG CGA GCG CTC CCA CCA TGA CGG-3´ |
| R13A/S16A-FP | 5´-ATG GTG GGA TCG CTC GCC AAG GAT GCG CTC AAT CAC AAG C-3´ |
| R13A/S16A-RP | 5´-GCT TGT GAT TGA GCG CAT CCT TGG CGAG CGA TCC CAC CAT-3´ |
| S16A/N18A-FP | 5´-GGG ATC GCT CCG CAA GGA TGC GCT CGC TCA CAA GCT GAT GAA G-3´ |
| S16A/N18A-RP | 5´-CTT CAT CAG CTT GTG AGC GAG CGC ATC CTT GCG GAG CGA TCC C-3´ |
| Y46A-FP | 5´- GGC GAC CTG CCG CAT GCC AAC GAC GAC CTT TG -3´ |
| Y46A-RP | 5´- CAA AGG TCG TCG TTG GCA TGC GGC AGG TCG CC -3´ |
| E77A-FP | 5'-gcg atc acg ccg gca tac aac cgc agc-3' |
| E77A-RP | 5'-gct gcg gtt gta tgc cgg cgt gat cgc-3' |
| E77K-FP | 5´-TCT GGC GAT CTT CAC GCC GAA GTA CAA CCG CAG CTA TC-3´ |
| E77K-RP | 5´-GAT AGC TGC GGT TGT ACT TCG GCG TGA TCG CCA GA-3´ |
| E77L-FP | 5'-ttc tgg cga tca cgc cgt tat aca acc gca gct atc-3' |
| E77L-RP | 5'-gat agc tgc ggt tgt ata acg gcg tga tcg cca gaa-3' |
| E77M-FP | 5'-gtt ctg gcg atc acg ccg atg tac aac cgc agc tat ccg-3' |
| E77M-RP | 5'-cgg ata gct gcg gtt gta cat cgg cgt gat cgc cag aac-3' |
| Y78A-FP | 5'- gcg atc acg ccg gaa gcc aac cgc agc tat cc -3' |
| Y78A-RP | 5'- gga tag ctg cgg ttg gct tcc ggc gtg atc gc -3' |
| N79A-FP | 5´- GCG ATC ACG CCG GAA TAC GCC CGC AGC TAT CCG -3´ |
| N79A-RP | 5´- CGG ATA GCT GCG GGC GTA TTC CGG CGT GAT CGC -3´ |
| R80E-FP | 5´-GAT CAC GCC GGA ATA CAA CGA AAG CTA TCC GGG CAT GAT CA-3´ |
| R80E-RP | 5´-TGA TCA TGC CCG GAT AGC TCT TGT TGT ATT CCG GCG TGA TC -3´ |
| R80K-FP | 5´-GAT CAC GCC GGA ATA CAA CAA GAG CTA TCC GGG CAT GAT CA-3´ |
| R80K-RP | 5´- TGA TCA TGC CCG GAT AGC TTT CGT TGT ATT CCG GCG TGA TC -3´ |
| R80L-FP | 5´-GCC GGA ATA CAA CCT CAG CTA TCC GGG CA -3´ |
| R80L-RP | 5´-TGC CCG GAT AGC TGA GGT TGT ATT CCG GC-3´ |
| R80M-FP | 5´- GAT CAC GCC GGA ATA CAA CAT GAG CTA TCC GGG CAT GAT CA -3´ |
| R80M-RP | 5´- TGA TCA TGC CCG GAT AGC TCA TGT TGT ATT CCG GCG TGA TC -3´ |
| R95A-FP | 5´-ATC GAC TGG GCC ACC GCC CCC TAT GGC CAG AAC-3´ |
| R95A-RP | 5´-GTT CTG GCC ATA GGG GGC GGT GGC CCA GTC GAT-3´ |
| R95E-FP | 5´-CAT CGA CTG GGC CAC CGA ACC CTA TGG CCA GAA CT-3´ |
| R95E-RP | 5´-AGT TCT GGC CAT AGG GTT CGG TGG CCC AGT CGA TG-3´ |
| S113A-FP | 5'-CCG CCG TCA TCG GCA CAG CCC CGG GCG TG-3' |
| S113A-RP | 5'-CAC GCC CGG GGC TGT GCC GAT GAC GGC GG-3' |
| G115I-FP | 5'- cgg cac atc gcc gat cgt gat cgg tgc g -3' |
| G115I-RP | 5'- cgc acc gat cac gat cgg cga tgt gcc g -3' |
| G115F-FP | 5'- cgg cac atc gcc gtt cgt gat cgg tgc g -3' |
| G115F-RP | 5'- cgc acc gat cac gaa cgg cga tgt gcc g -3' |

**Table S1 The mutagenic oligonucleotide primers for site-directed mutagenesis of FerB His_6_.**

^*^the underlined nucleotides represent the mutated codons
