# Supplementary material for: The Structural and Functional Basis of Catalysis Mediated by NAD(P)H:acceptor Oxidoreductase (FerB) of Paracoccus denitrificans
Source: PLoS One. 2014 May 9;9(5):e96262. doi: 10.1371/journal.pone.0096262 (PMC4015959; doi:10.1371/journal.pone.0096262)
Supplement: Table S2 — Data collection from SAXS. (DOC) [file pone.0096262.s002.doc]

**Table S2 Data collection from SAXS.**

| **Data-collection parameters** |  |  |  |
| --- | --- | --- | --- |
| Instrument | BioSAXS-1000 (Rigaku) |  |  |
| Wavelength (Å) | 1.5418 |  |  |
| *q* range (Å^-1^) | 0.008–0.65 |  |  |
| Exposure time (min) | 30 |  |  |
| Temperature (K) | 290 |  |  |
| Concentration range (mg/ml) | 5.0 | 2.5 | 1.25 |
| **Structural parameters** |  |  |  |
| *I*(0) (A.U.) [from Guinier] | 0.07 | 0.07 | 0.06 |
| *R_g_*(Å) [from Guinier] | 26.8 ± 1.5 | 27.2 ± 1.4 | 23.8 ± 2.8 |
| *I*(0) (A.U.) [from P(r)] | 0.07 | 0.07 | 0.07 |
| *R_g_*(Å) [from P(r)] | 26.7 | 26.6 | 26.2 |
| *D*_max_ (Å) | 90 | 95 | 80 |
| Porod volume estimate | 144 000 | 132 000 | 120 000 |
| Dry volume calculated from sequence (Å^3^) FerBHis_6_-dimeric/tetrameric | 51488/102955 |  |  |
| **Software employed** |  |  |  |
| Primary data reduction | SAXSLab*2.0.1b2* |  |  |
| Data processing | PRIMUS |  |  |
| Computation of model intensities | CRYSOL |  |  |
| Oligomeric state | OLIGOMER |  |  |
