# Supplementary material for: The Structural and Functional Basis of Catalysis Mediated by NAD(P)H:acceptor Oxidoreductase (FerB) of Paracoccus denitrificans
Source: PLoS One. 2014 May 9;9(5):e96262. doi: 10.1371/journal.pone.0096262 (PMC4015959; doi:10.1371/journal.pone.0096262)
Supplement: Table S3 — Amino-acid identity and structural similarity across the structural homologs of FerB in a FMN reductase protein family (PF03358). (DOCX) [file pone.0096262.s003.docx]

**Table S3 Amino-acid identity and structural similarity across the structural homologs of FerB in a FMN reductase protein family (PF03358).**

| PDB  Code | UniProt  ID | Protein name | Organism | Z-score  (DALI) | Sequence  Identity (%) |
| --- | --- | --- | --- | --- | --- |
| 3U7R | A1B9E3 | FerB | *Paracoccus denitrificans* | 37.7 | 100 |
| 3SVL | P0AGE6 | ChrR | *Escherichia coli* | 24.2 | 35 |
| 3S2Y | D5QFC5 | Gh-ChrR | *Gluconacetobacter hansenii* | 24.1 | 35 |
| 1RTT | Q9I4D4 | T1501 | *Pseudomonas aeruginosa* | 23.2 | 34 |
| 2Q62 | Q92R45 | ArsH | *Sinorhizobium meliloti* | 20.6 | 17 |
| 2FZV | Q7UC03 | ArsH | *Shigella flexneri* | 20.4 | 17 |
| 3GFR | O07529 | YhdA | *Bacillus subtilis* | 19.1 | 20 |
| 1T0I | Q07923 | YLR011wp | *Saccharomyces cerevisiae* | 17.6 | 21 |
| 2VZH | Q9F9T2 | EmoB | EDTA-degrading bacterium BNC1 | 17.4 | 21 |
| 2OYS | Q97NR6 | Sp1951 | *Streptococcus pneumoniae* | 17.1 | 14 |
| 1ZWL | Q9I509 | WrbA | *Pseudomonas aeruginosa* | 14.4 | 16 |
| 2RG1 | P0A8G6 | WrbA | *Escherichia coli* | 13.5 | 14 |
