# Supplementary figures and images for: The Structural and Functional Basis of Catalysis Mediated by NAD(P)H:acceptor Oxidoreductase (FerB) of Paracoccus denitrificans
Source: PLoS One. 2014 May 9;9(5):e96262. doi: 10.1371/journal.pone.0096262 (PMC4015959; doi:10.1371/journal.pone.0096262)

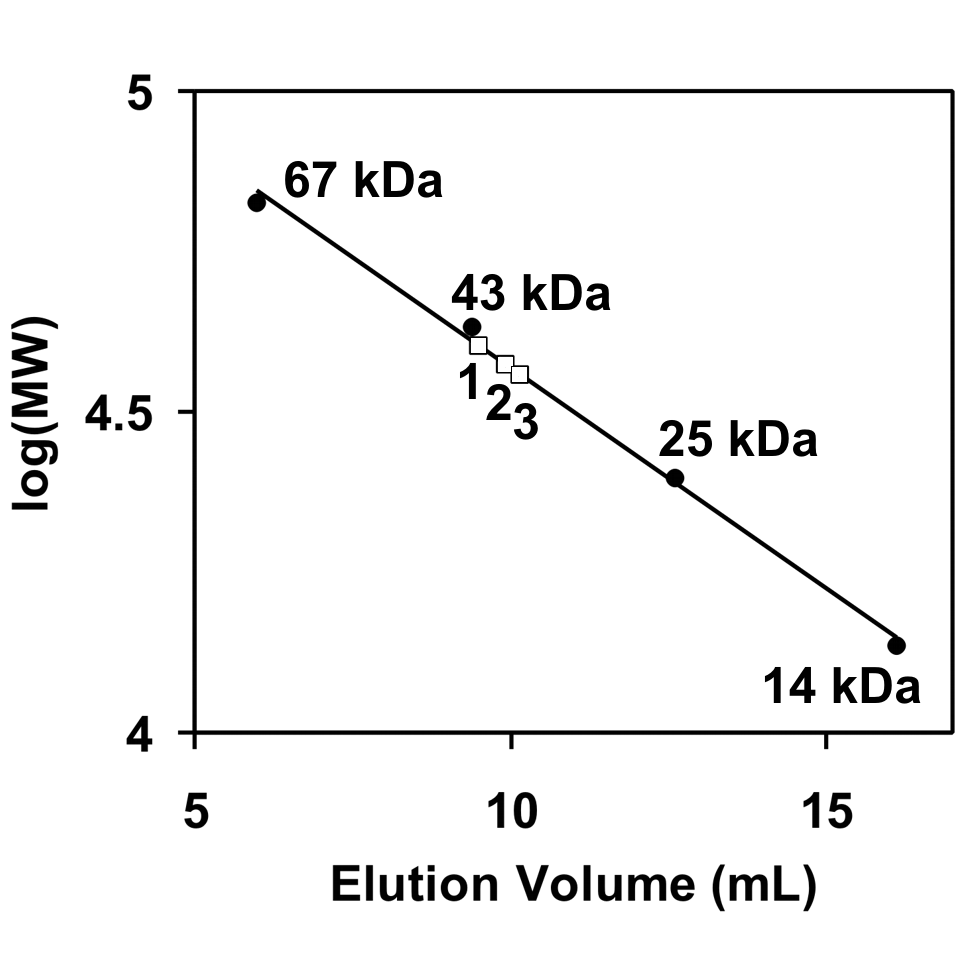

Supplement: Figure S1 — Superose 12 analytical size exclusion chromatography of FerBHis6 holoenzyme (1), apoenzyme (2), and FerBHis6-R95E mutant (3). The calibration curve used to estimate the native molecular weight based on the elution position is indicated. (TIF) [file pone.0096262.s004.tif]

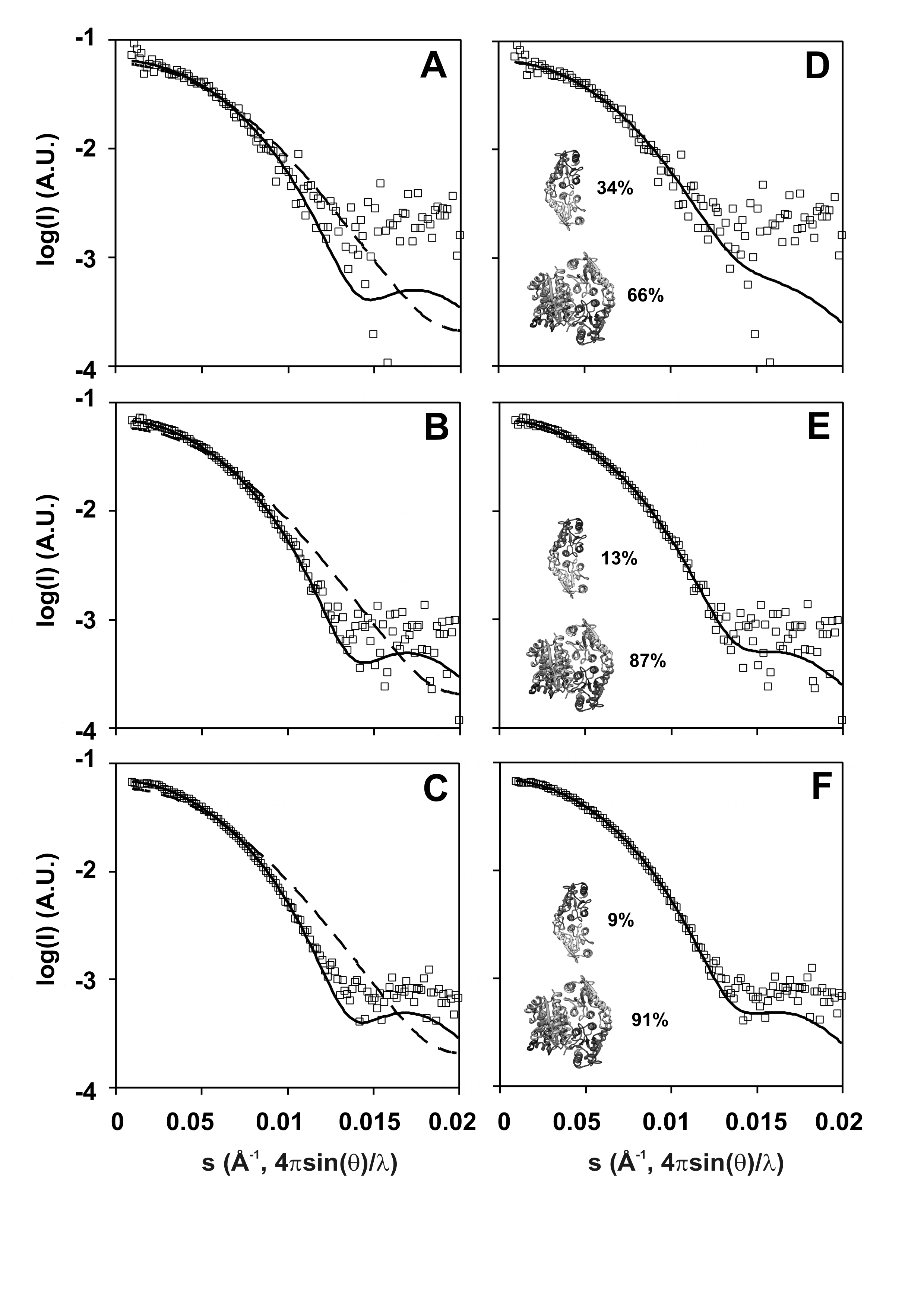

Supplement: Figure S2 — SAXS scattering curves. X-ray scattering of FerBHis6 was compared with calculated scattering of the dimer (dashed line) and the tetramer (solid line) computed by CRYSOL for concentrations 1.25 (A), 2.5 (B) and 5.0 (C) mg/mL. The program OLIGOMER was used to estimate dimer and tetramer volume fractions for concentrations 1.25 (D), 2.5 (E) and 5.0 (F) mg/ml. (TIF) [file pone.0096262.s005.tif]

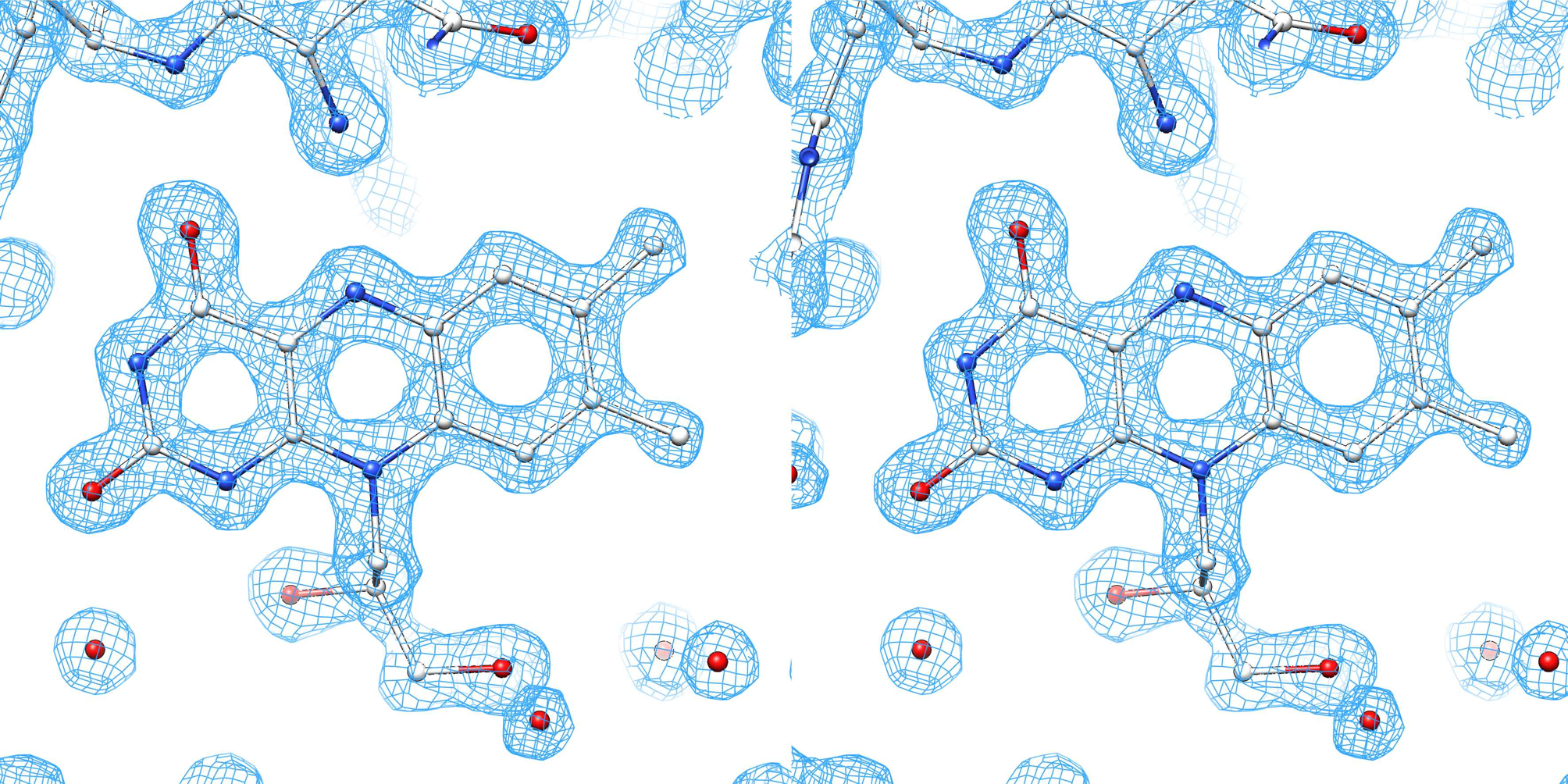

Supplement: Figure S3 — Electron density map of FMN. Stereo view of the 2F0 – Fc electron density map contoured at 1.0 σ cut-off corresponding to FMN at the active site. (TIF) [file pone.0096262.s006.tif]

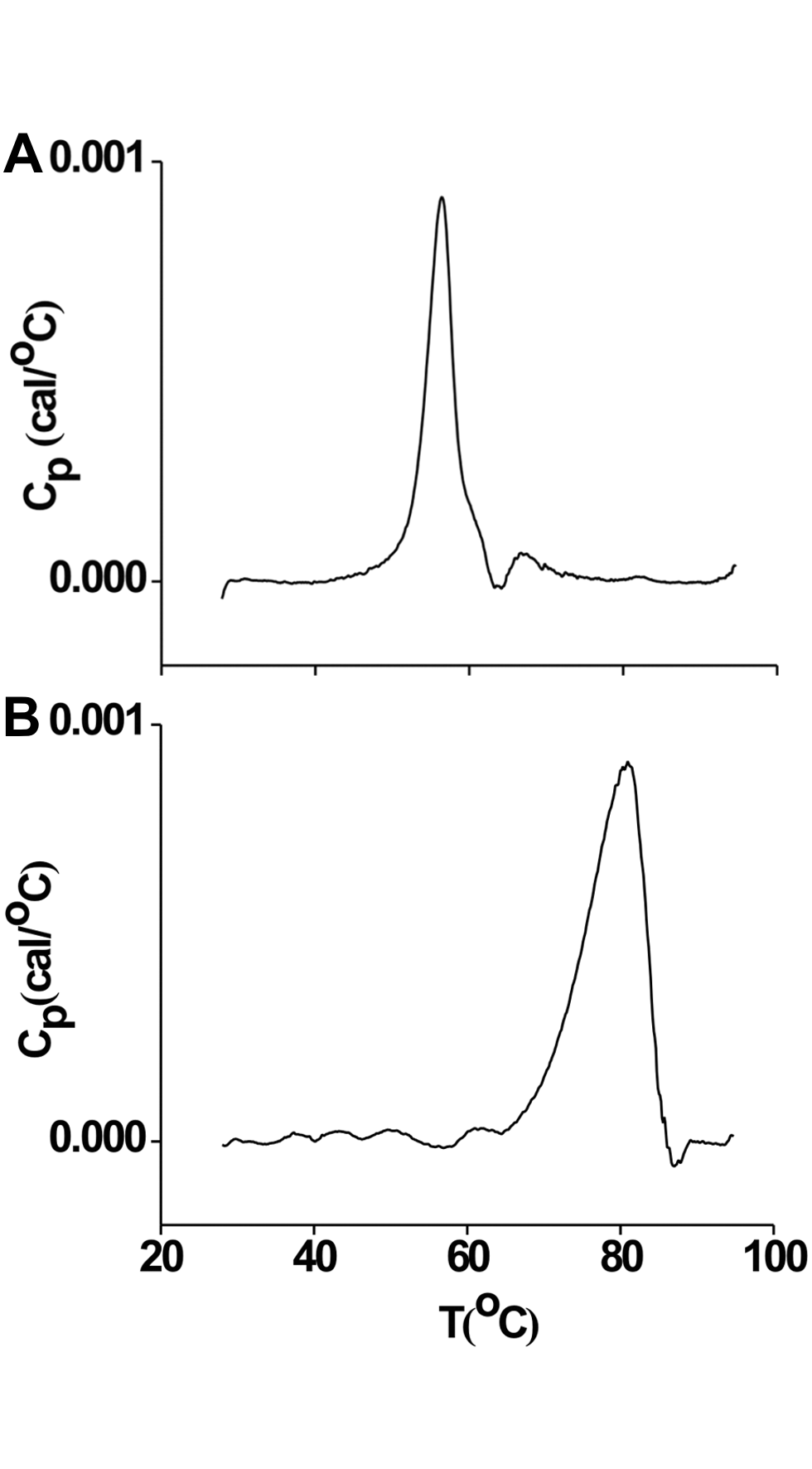

Supplement: Figure S4 — Thermal stability of apoFerBHis6 (A) and FerBHis6 (B) followed by DSC. Protein concentrations were adjusted to 4.3 mg/mL for apoFerBHis6 and 2.5 mg/mL for FerBHis6 in buffer C and data were collected from 25 to 95 °C at a heating rate of 60 °C per hour. (TIF) [file pone.0096262.s007.tif]

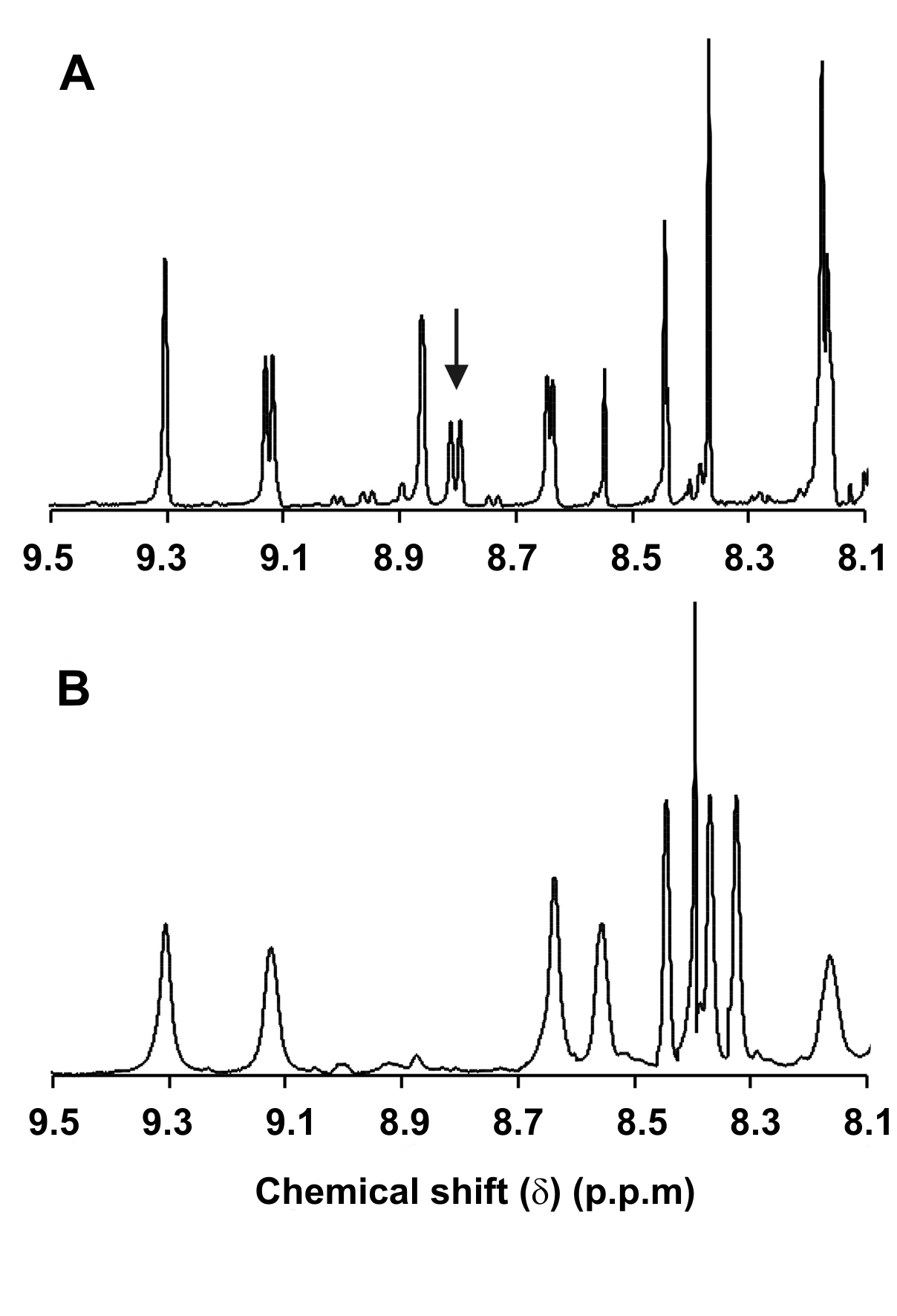

Supplement: Figure S5 — NMR spectra of NAD+. The 1H NMR aromatic region of NAD+ formed from [4B-2H]NADH (A) or [4A-2H]NADH by FerBHis6 in the presence of 1,4-benzoquinone showing a resonance signal at 8.8-8.9 p.p.m. (A) or no resonance signal (B). The enzyme removed the proton from B-side (pro-S, si-face) of the dihydrogennicotinamide ring. (TIF) [file pone.0096262.s008.tif]

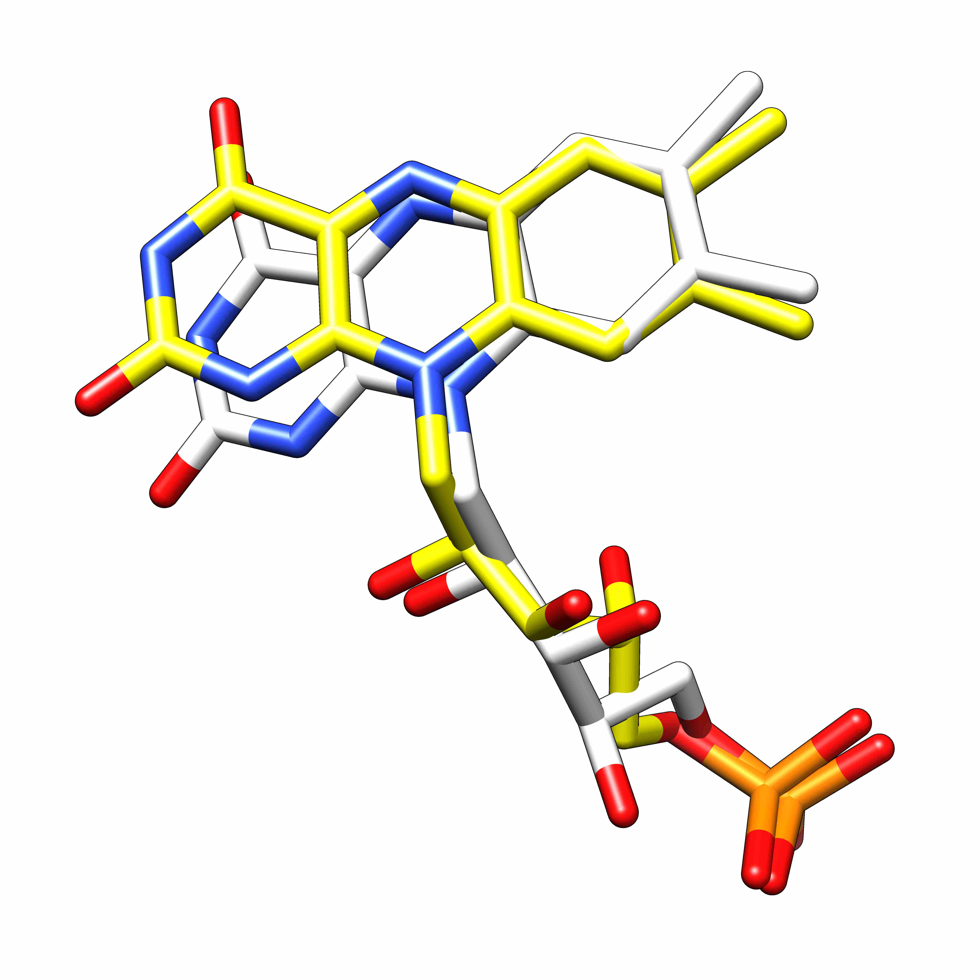

Supplement: Figure S6 — Superimposition of FMN from the crystal structure of FerBHis6 (white) and from the docking-derived complex (yellow). (TIF) [file pone.0096262.s009.tif]

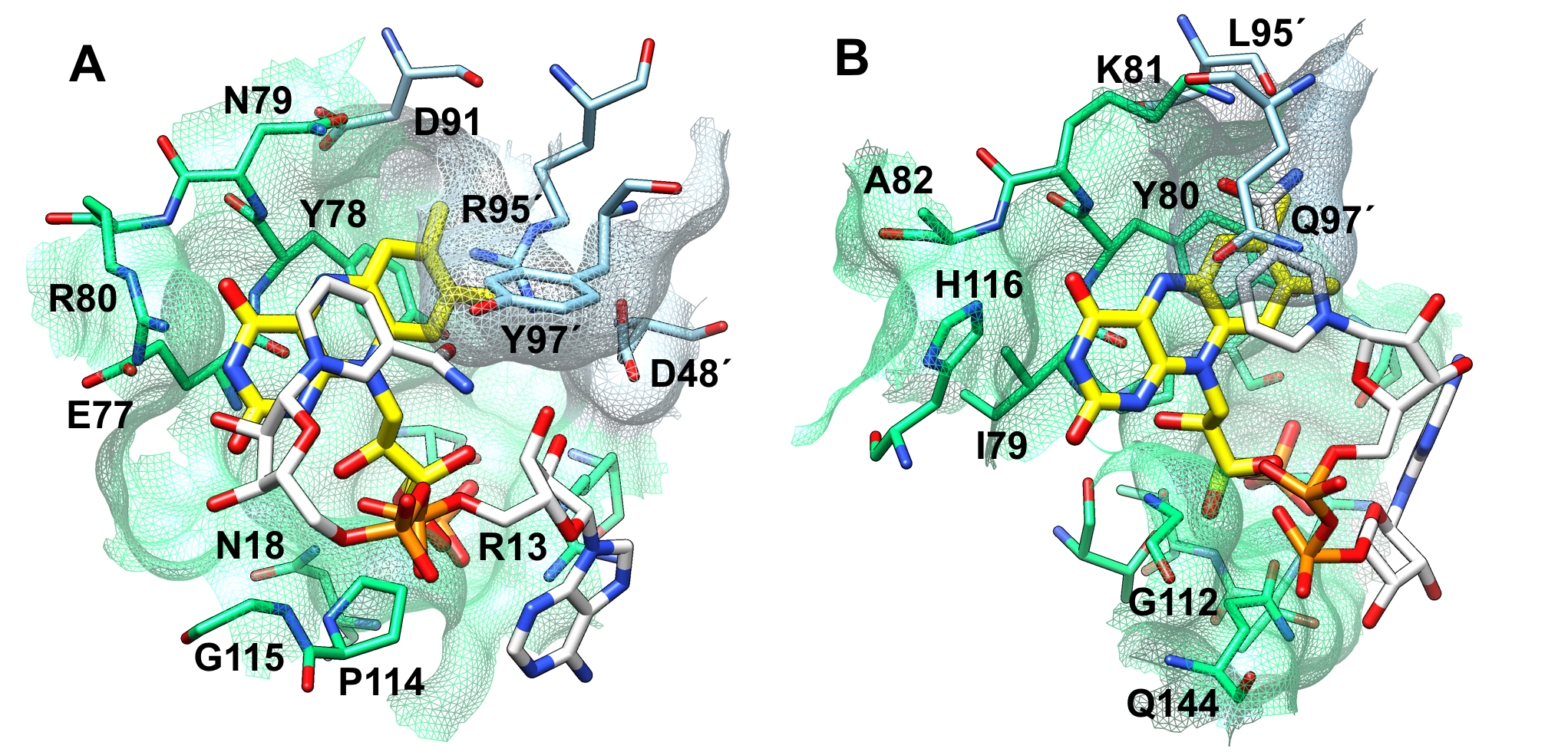

Supplement: Figure S7 — Structure of the docked FerBHis6-NADH complex. NADH was modeled into the FerBHis6 structure using AutoDock Vina v1.1.2. FMN and NADH are shown with yellow and white carbons, respectively (A). The nicotinamide ring was stacked on top of the isoalloxazine ring at distance 3.8 Å that corresponding with distances of other NADH-dependent FMN reductases. The residues are depicted in green and violet to represent belonging to two different subunits. The known crystal structure of EmoB complex with NADH (PDB ID: 2VZJ) is included for sake of comparison (B). (TIF) [file pone.0096262.s010.tif]
